# Cohort Profile Update: The Lothian Birth Cohorts of 1921 and 1936

**DOI:** 10.1093/ije/dyy022

**Published:** 2018-03-12

**Authors:** Adele M Taylor, Alison Pattie, Ian J Deary

**Affiliations:** 1 Department of Psychology, University of Edinburgh, Edinburgh, UK; 2 Centre for Cognitive Ageing and Cognitive Epidemiology, University of Edinburgh, Edinburgh, UK

## The original cohort

The original Lothian Birth Cohorts of 1921 (LBC1921) and 1936 (LBC1936) were designed as follow-up studies to the Scottish Mental Surveys of 1932 (SMS1932) and 1947 (SMS1947), respectively.^1^ The SMS1932 took place simultaneously across schools in Scotland on 1 June 1932, and used the Moray House Test (No. 12; MHT) of general intelligence. Almost every child attending school and born in 1921 (*N* = 87 498) was tested.^2^ The same MHT was administered to almost every child born in 1936 and attending school on 4 June 4 1947 for the SMS1947 (*N* = 70 805).^3^ As described in the Cohorts Profile published in 2012,^4^ decades later, participants of both Surveys, mostly living in Edinburgh and the surrounding area (the Lothians) in older age, were invited to participate in the Lothian Birth Cohort (LBC) studies. Between 1999 and 2001, 550 of the SMS1932 were recruited to Wave 1 of the LBC1921 study, at a mean age of 79 years. Between 2004 and 2007, 1091 members of SMS1947 were recruited to Wave 1 of the LBC1936 study, at a mean age of 70 years. Both cohorts re-sat the MHT at initial follow-up. In addition, a large amount of other cognitive, psychosocial, lifestyle, medical, biomarker, genetic, brain imaging and other data were collected. There are baseline protocol articles for LBC1921^5^ and LBC1936,^6^ and a separate baseline brain imaging protocol article for LBC1936.^7^

## What is the reason for the new data collection?

There are two main reasons for the Profile Update. First, there have been new waves of data collection since the LBCs’ profiles were reported.^4^ Second, there have been many new types of data collected.

### New waves of data collection

The LBC studies set out principally to examine the nature and determinants of non-pathological cognitive ageing from childhood to older age, and within in older age.^5^ Waves of testing have been conducted at roughly 3-yearly intervals since inception: the LBC1921 have been followed up five times, from age 79 to age 92 years;^8–11^ and the LBC1936 have been followed up four times from age 70 to age 79 years.^6^^,^^9^^,^^11^ A fifth wave of the LBC1936 study started in autumn 2017 and tests the LBC1936 participants in their early 80s. New waves of data collection since the LBCs’ profiles were published in 2012 (LBC1921 Waves 4 and 5; LBC1936 Waves 3 and 4) have been conducted during key periods of ageing, where the risk of onset of cognitive impairment and dementia is increased. Consequently, the scope of the studies has extended to identifying more risk and protective factors that have the potential to be interventions to reduce the risk of cognitive loss in later life. Although the major focus remains on cognitive and brain outcomes, advancements in knowledge and technology since 2012—and the diverse range of data collected from the participants—have resulted in opportunities for new variables and data collection methods to be adopted, and have allowed for aspects of ageing to be examined beyond those for which core grant funding was obtained.

### New types of data collected

A major reason for the profile update is the huge amount of new types of data collected on the LBCs since their profile^4^ was published, especially: whole-genome sequencing, longitudinal DNA methylation, longitudinal gene expression, lipidomics, post-mortem brain tissue, induced pluripotent stem cells (iPSC), inflammatory markers, oxidative stress markers, life course geographical information, objectively measured physical activity and sedentary behaviour, and dementia ascertainment.

## What will be the new areas of research?

These correspond to the two reasons for the profile update listed in the previous section. First, the availability of five waves of consistently measured cognitive, biomedical, brain imaging, psychosocial, lifestyle and other data in the LBC1921 and four waves of such data in the LBC1936 mean that longitudinal analyses are possible, covering the age ranges 79-92 and 70-79, respectively. The numbers of waves means that non-linear effects can now be explored, and that the combined cohorts’ data cover most of the period we think of as older age in humans.

The last paragraph of the previous section makes clear the new types of exposures, outcomes and experimental models that are newly possible with the LBCs’ updated data. Thus, for example: longitudinal methylomics and transcriptomics research is now possible; geographical exposures such as green space and pollution are available as life course exposures; and the studies can be extended from their previous focus on non-pathological cognitive ageing to dementias. The bank of cells for pluripotent stem cell formation (some have already been re-programmed) are available for experimental research on ageing, backed up with rich phenotyping and genotyping. The growing brain tissue bank allows a wide range of research on post-mortem tissue, linked with the phenotypic and genetic data. There is a protocol paper for the post-mortem brain tissue collection, preparation and experimental work.^12^

Wave 4 of the LBC1936 was timed carefully, so that the participants had the same mean and standard deviation (SD) of age as the LBC1921 participants at Wave 1. By luck, the numbers of participants are identical, *N* = 550 for each (i.e. LBC1921 at Wave 1 and LBC1936 at Wave 4). This provides new possibilities for studying cohort effects on ageing, because the same data have been collected on people of the same age, but who were born 15 years apart.

## Who is in the cohort?

**Figure 1:**
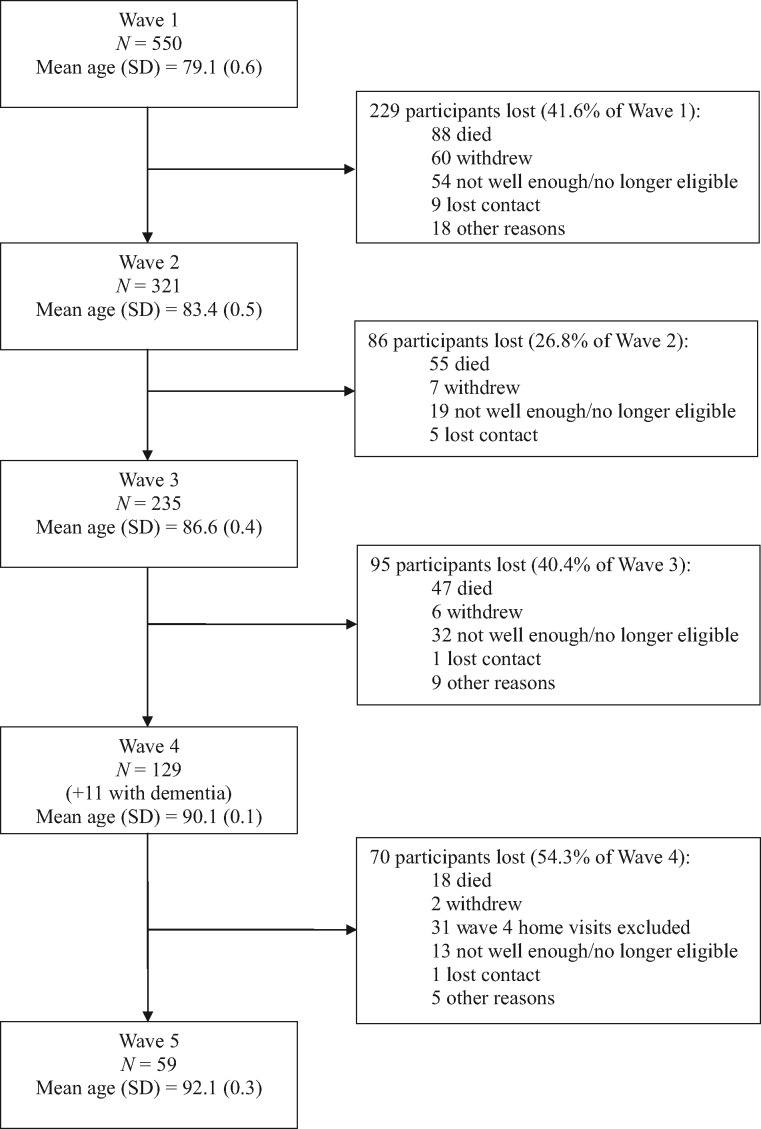
Waves of testing and attrition between waves in the LBC1921 study.

**Figure 2:**
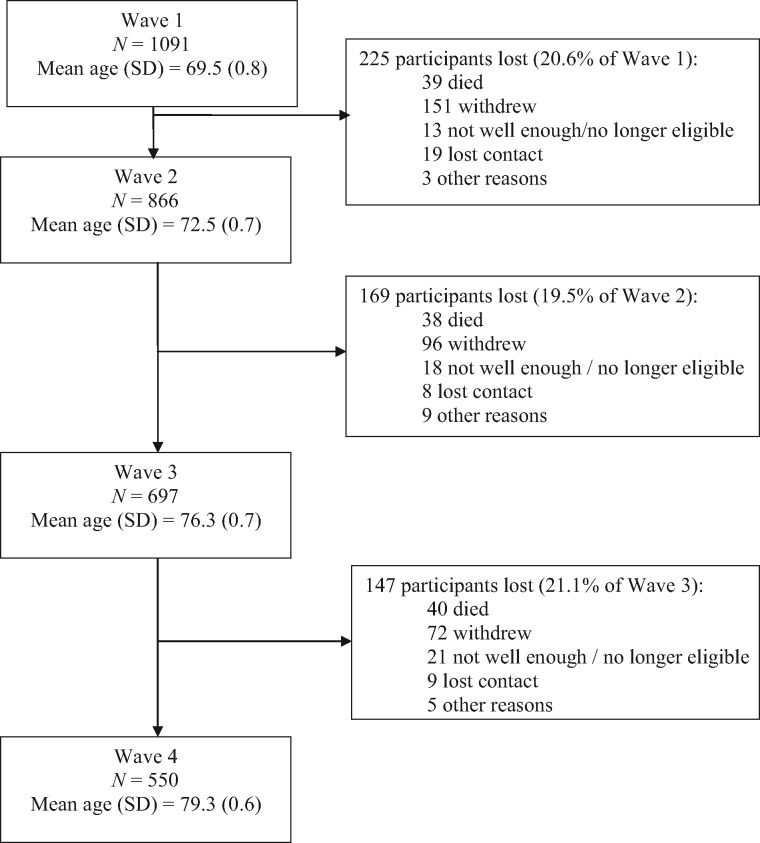
Waves of testing and attrition between waves in the LBC1936 study.

Table 1 and Table 2 summarize the waves of assessment for LBC1921 and LBC1936, with details of mean age, numbers, sample composition and data types collected at each wave. Flowcharts, with numbers of participants tested and reasons for attrition at each wave, are presented in Figure 1 (for LBC1921) and Figure 2 (for LBC1936). Of the original 550 LBC1921 participants, 59 ‘completers’ have participated in all waves, including the most recent follow-up at mean age 92 (Wave 5) between April and December of 2013. Of the original 1091 LBC1936 participants, 550 ‘completers’ have participated in all waves, including the most recent follow-up at mean age 79 (Wave 4) between November 2014 and February 2017. Only a small number of participants in each cohort have missed a wave of testing but returned for subsequent waves (maximum *n* = 11). Rates of attrition between waves in the LBC1936 have been consistently ∼20%, though in the older LBC1921 cohort they have varied from ∼27% to 54%. For both cohorts, between most waves, the major causes of attrition have been death (up to 64% of non-attendees) and permanent withdrawal resulting from ill health and chronic incapacity (up to 67% of non-attendees).

**Table 1:** The Lothian Birth Cohort of 1921 (LBC1921)

Variable measured	Wave 1	Wave 2	Wave 3	Wave 4	Wave 5
	1999–2001	2003–05	2007–08	2011–12	2013
	79.1 years (SD = 0.6)	83.4 years ( SD = 0.5)	86.6 years (SD = 0.4)	90.1 years (SD = 0.1)	92.1 years (SD = 0.3)
	*N* (m/f) = 550	*N* (m/f) = 321	*N* (m/f) = 237	*N* (m/f) = 140	*N* (m/f) = 59
	(234/316)	(145/176)	(109/128)	(62/78)	(26/33)
Cognitive ability					
Moray House Test No. 12					
Cognitive test battery (executive function, reasoning and memory)					
Processing speed measures					
Social and demographic data					
Includes: residence, childhood overcrowding/deprivation, education, occupation					
Marital status					
Questionnaires					
Includes: family, loneliness, social support, activities, well-being, personality					
Includes: children, employment, religious activity, spiritual well-being					
Neighbourhood questions, Life Orientation Test-Revised, Sense of Coherence					
Warwick-Edinburgh Mental Wellbeing Scale					
The Brief Resilience Scale, Attitudes to Ageing Questionnaire, Life review					
Physical and medical					
Various including: smoking, alcohol, physical fitness, disease history, medication, blood pressure, height, weight					
Visual acuity					
Demi-span, dentition, electrocardiogram					
Head circumference					
Facial symmetry analysis					
Fluctuating asymmetry, ankle-brachial pressure index					
Self-rated health					
Hand asymmetry analysis, musculoskeletal history, hearing					
Family history of disease					
Biochemistry, haematology, inflammation and oxidative stress					
Various including full blood count, coagulation screening and glycated haemoglobin					
Triglyceride, total serum cholesterol					
Vitamin B_12_, red cell folate, thyroid stimulating hormone, thyroxine					
Serum folate,					
PT ratio, APTT ratio, D-Dimer, urea, estimated glomerular filtration rate, C-reactive protein					
Sodium, potassium, albumin					
Tumour necrosis factor alpha					
Von Willebrand Factor, interleukin 6					
Trolox equivalent antioxidant* *capacity, DNA damage, antioxidant vitamins					
Genetic analysis					
Apolipoprotein E genoptyping, genome-wide genotyping, whole-genome sequencing					
Telomere length					
DNA methylation					
Brain magnetic resonance imaging (MRI)					
Structural brain MRI measures (various; sub-sample *n* = 42)					
Diffusion tensor derived long-range tract variables, volume measures, visual atrophy and lesion rating, Desikan-Killiany Atlast Parcellation (FreeSurfer), neuroradiological report					
Retinal imaging					
Microvascular widths and branching geometry					
Linkages					
Date and cause of death, dementia diagnosis					
Morbidity					

Participants of LBC1921 were originally tested on the Moray House Test No.12 intelligence test as part of the Scottish Mental Survey 1932 (*N* = 87 498) at a mean age of 10.9 years (SD = 0.3).

Table key:


 = data available at time of previous cohorts profile publication (2012).


 = new wave of data, repeat collection of previous variable.


 = new variable not previously collected.

PT ratio, prothrombin ratio; APTT, activated partial thromboplastin time ratio; m/f, male/female.

**Table 2:** The Lothian Birth Cohort of 1936 (LBC1936)

Variable measured	Wave 1	Wave 2	Wave 3	Wave 4	Wave 5^a^
	2004–07	2007–10	2011–13	2014–17	2017–19
	69.5 years (SD = 0.8)	72.5 years (SD = 0.7)	76.3 years (SD = 0.7)	79.3 years (SD = 0.6)	∼82 years
	*N* (m/f) = 1091	*N* (m/f) = 866	*N* (m/f) = 697	*N* (m/f) = 550	*N* = ∼440
	(548/543)	(448/418)	(360/337)	(275/275)	
Cognitive ability					
Moray House Test No. 12					
Cognitive test battery					
‘Frontal’ function tests (sub-sample *n* = 90)					
Test of Premorbid Functioning, Trail Making Test (Part B)					
Raven’s Progressive Matrices					
Financial literacy and competence					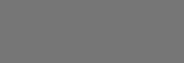
Social and demographic data					
Various including: age, marital status, residence					
Various including: deprivation, education, occupation, retirement					
Current employment status, volunteering and caring					
Questionnaires					
Various including: personality, quality of life, social support					
Various including: personality, loneliness					
Physical activity level, sport/exercise, intellectual/social activities					
Food Frequency Questionnaire					
Various including: social support, satisfaction with life, well-being					
Sense of coherence, personal and social well-being					
Attitudes to Ageing Questionnaire, health literacy, bilingualism					
Various including: lifetime addresses, lifetime activity					
The Brief Resilience Scale, self-reported memory problems					
Sleep quality and pattern					
Various including: apathy, sedentary patterns, social desirability					
Musical experience and expertise					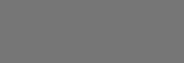
Physical and medical					
Various including: smoking, alcohol, disease history, medication, height, weight, visual acuity, blood pressure, physical fitness					
Self-rated health, dentition, stand tests					
Head circumference, menopause					
Ankle-brachial pressure index					
Facial symmetry photographs					
Salt intake, bioelectrical impedance analysis					
Hearing					
Incidence of falls					
Sedentary behaviour analysis by electronic activity monitor					
Biochemistry and haematology analysis					
Various including: full blood count, coagulation, cholesterol					
Various including: D-Dimer, eGFR, iron, urine analysis					
Red cell folate					
Urate					
Sex hormone-binding globulin, testosterone					
Free androgen index					
International normalized ratio warfarin					
Inflammation markers					
High-sensitivity CRP, Von Willebrand Factor, interleukin 6					
Various including: cystatin C, lipoprotein A, vitamin D					
Oxidative stress					
Urinary-8 isoprostane, trolox equivalent antioxidant* *capacity					
DNA damage, antioxidant vitamins					
Blood brain barrier					
S100 beta					
Saliva analysis					
DNA (if unable to obtain blood sample only)					
Cortisol (sub-sample *n* = 90)					
Immunology					
Cytomegalovirus (CMV) serostatus and titre					
iPSC stem cell reprogramming					
*P*eripheral blood mononuclear cell samples taken					
Brain tissue analysis					
Pre-mortem consent					
Genetic analysis					
Telomere length					
Apolipoprotein E genotyping, genome-wide genotyping					
DNA methylation					
Transcriptome-wide gene expression					
Whole-genome sequencing					
Brain magnetic resonance imaging (MRI)					
Various including: DT-tracts, volumes, atrophy/lesion rating					
Whole brain structural connectome					
Carotid Doppler ultrasound					
Velocities, stenosis, intima-media thickness					
Retinal imaging					
Microvascular branching geometry, fractal dimension, tortuosity					
Linkages					
Date and cause of death, hospital discharge diagnosis					
Dementia diagnosis					
Medical prescription data					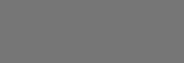

Participants of LBC1936 were originally tested on the Moray House Test No.12 intelligence test as part of the Scottish Mental Survey 1947 (*N* = 70 805) at a mean age of 10.9 years (SD = 0.3).

Table key

Data available:


 = data available at previous cohort profile publication (2012).


 = new data wave, repeat of previous variable.


 = new variable not previously collected.

Forthcoming data:


 = planned for Wave 5, repeat of previous variable.

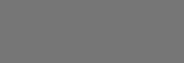
 = new variable forthcoming, not previously collected.

a Wave 5 in progress from October 2017: age and *N* are estimates based on attrition at previous waves.

CRP, C-reactive protein; eGFR, estimated glomerular filtration rate; m/f, male/female.

With regard to how attrition affects key variables, the differences between the tested sample at each wave and those who dropped out between waves of each study are shown for the first time in Table 3 (LBC1921) and Table 4 (LBC1936). By wave, compared with those who remained in the LBC1921 study at Waves 1 to 5, participants who dropped out had: significantly lower older-age IQ scores at Wave 1 (*P* < 0.001), Wave 2 (*P* = 0.004) and Wave 3 (*P* = 0.02); lower Mini-Mental State Examination (MMSE) scores at Waves 1–4 (all *P*-values < 0.01); and weaker grip strength at Waves 1 (*P* = 0.007) and 2 (*P* = 0.002). By wave, compared with those who remained in the LBC1936 study at Waves 1 to 4, participants who dropped out had: significantly lower older-age IQ scores at Wave 1 (*P* < 0.001), Wave 2 (*P* = 0.001) and Wave 3 (*P* = 0.003); lower MMSE scores at Wave 1 (*P* = 0.04), Wave 2 (*P* < 0.001) and Wave 3 (*P* = 0.001); lower socioeconomic status, represented by less professional occupational types, at Wave 1 (*P* = 0.001), Wave 2 (*P* = 0.04) and Wave 3 (*P* < 0.001); and lower physical fitness, as assessed by lung function at Wave 1 (*P* < 0.001), Wave 2 (*P* = 0.02) and Wave 3 (*P* = 0.005) and by grip strength at Wave 1 (*P* = 0.003).

**Table 3:** Sample characteristics at each wave by returning status in the Lothian Birth Cohort 1921

Variable	Wave 1	Dropout W1	Wave 2	Dropout W2	Wave 3	Dropout W3	Wave 4	Dropout W4	Wave 5
	(*N* = 550)	(*N* = 231)	(*N* = 321^c^)	(*N* = 88)	(*N* = 235^d^)	(*N* = 106)	(*N* = 129)	(*N* = 70)	(*N* = 59)
Sex (male)	42.5%	39%	45.2%	40.4%	46.4%	47.2%	45.7%	47.1%	44.1%
Age-11 IQ	100.0 (15.0)	98.6 (15.6)	101.0 (14.5)	99.4 (15.1)	101.6 (14.2)	99.9 (15.2)	103.0 (13.2)	104.3 (13.9)	101.4 (12.2)
Age-79 IQ	100.0 (15.0)	96.8 (16.3)	102.3 (13.6)	98.7 (14.5)	103.7 (12.9)	101.3 (14.3)	105.5 (11.4)	106.6 (11.0)	104.3 (11.7)
MMSE	28.2 (1.69)	27.9 (1.92)	28.1 (1.94)	27.4 (2.40)	27.8 (2.31)	27.1 (2.91)	27.3 (2.93)	26.7 (3.64)	27.2 (2.36)
Father’s SES	2.73 (0.93)	2.80 (0.92)	2.69 (0.94)	2.72 (0.84)	2.68 (0.97)	2.70 (0.79)	2.66 (1.10)	2.53 (1.01)	2.81 (1.18)
Participant SES	2.24 (0.88)	2.37 (0.94)	2.15 (0.83)	2.26 (0.80)	2.11 (0.83)	2.20 (0.75)	2.04 (0.89)	1.90 (0.81)	2.20 (0.96)
CVD(yes)^a^	29.5%	28.6%	15.9%	13.5%	20.0%	18.9%	45%	45.7%	50.8%
Grip strength^b^	26.5 (9.11)	25.3 (8.42)	24.9 (9.19)	22.5 (8.00)	21.5 (8.77)	20.2 (8.40)	20.3 (8.16)	19.7 (8.58)	17.9 (9.11)
FEV_1_	1.88 (0.62)	1.77 (0.63)	–	–	1.77 (0.56)	1.70 (0.52)	1.66 (0.55)	1.62 (0.57)	1.53 (0.54)

Values given are mean and standard deviation, except for sex and CVD, for each wave sample split by returning status: Dropout W1 = completed Wave 1 only; Dropout W2 = completed Waves 1 and 2 only; Dropout W3 = completed Waves 1, 2 and 3 only; Dropout W4 = completed Waves 1-4 only. IQ calculated from Moray House Test score corrected for age in days at time of testing and converted to IQ scale. Father’s SES is participant’s father’s social class when the participant was aged 11 [five categories from professional (1) to unskilled labour (5)].

MMSE, Mini-Mental State Examination; SES, socioeconomic status; CVD, cardiovascular disease. FEV_1_, forced expiratory volume from the lungs in 1 s.

a CVD includes unsure and angina only at Wave 1.

b Grip strength reported as best of three from the right hand.

c Wave 2 includes two participants who did not attend Wave 1.

d Wave 3 includes two participants who attended Wave 1 but not Wave 2.

**Table 4:** Sample characteristics at each wave by returning status in the Lothian Birth Cohort 1936

Variable	Wave 1	Dropout W1	Wave 2	Dropout W2	Wave 3	Dropout W3	Wave 4
	(*N* = 1091)	(*N* = 225)	(*N* = 866)	(*N* = 169)	(*N* = 697)	(*N* = 158)	(*N* = 550^b^)
Sex (male)	50.2%	44.4%	51.7%	52.1%	51.6%	55.7%	50%
Age-11 IQ	100.0 (15.0)	99.8 (14.4)	100.0 (15.1)	99.3 (16.1)	100.2 (14.9)	100.0 (14.9)	100.0 (15.0)
Age-70 IQ	100.0 (15.0)	96.1 (16.9)	101.0 (14.3)	97.6 (16.0)	101.8 (13.8)	98.7 (14.6)	102.5 (13.7)
MMSE	28.8 (1.43)	28.6 (1.57)	28.8 (1.42)	28.3 (1.76)	28.7 (1.70)	28.2 (2.11)	28.5 (2.16)
Father’s SES	2.91 (0.94)	2.87 (0.94)	2.92 (0.94)	3.02 (0.95)	2.89 (0.94)	2.94 (0.89)	2.88 (0.95)
Participant SES	2.40 (0.91)	2.58 (0.86)	2.36 (0.92)	2.49 (0.88)	2.33 (0.93)	2.59 (0.90)	2.26 (0.92)
CVD (yes)	24.6%	27.6%	28.9%	30.2%	33.9%	32.3%	37.1%
Grip strength^a^	29.0 (10.1)	27.2 (10.6)	28.7 (9.45)	27.5 (9.40)	27.7 (10.1)	27.4 (11.0)	26.1 (9.43)
FEV1	2.36 (0.69)	2.15 (0.67)	2.30 (0.68)	2.19 (0.64)	2.11 (0.64)	1.98 (0.62)	2.11 (0.64)

Values given are mean and standard deviation, except for sex and CVD, for each wave sample split by returning status: Dropout W1 = completed Wave 1 only; Dropout W2 = completed Waves 1 and 2 only; Dropout W3 = completed Waves 1, 2 and 3 only. IQ calculated from Moray House Test score corrected for age in days at time of testing and converted to IQ scale. Father’s SES is participant’s father’s social class when the participant was aged 11 [five categories from professional (1) to unskilled labour (5)].

MMSE, Mini-Mental State Examination; SES, socioeconomic status; CVD, cardiovascular disease; FEV_1_, forced expiratory volume from the lungs in 1s.

a Grip strength reported as best of three from the right hand.

b Wave 4 includes 11 participants who did not attend Wave 3.

Data for completers only, at baseline and all subsequent waves, are given in Supplementary Table 1 (LBC1921) and Supplementary Table 2 (LBC1936), available as Supplementary data at *IJE* online. Comparison of scores from completers and those who dropped out at each wave showed that LBC1921 completers scored higher at baseline, and consistently across Waves 1 to 4, on the MMSE (all *P*-values < 0.01), and higher for physical fitness as assessed by lung function at Wave 1 (*P* = 0.003). LBC1936 completers scored higher at baseline, and consistently across Waves 1 to 3, on the MMSE (all *P*-values < 0.01), and higher for physical fitness as assessed by grip strength at Wave 1 ( *P* = 0.03) and by lung function at Waves 1 to 3 (all *P* -values < 0.01).

## What has been measured?

The core data collected (cognitive, medical, brain imaging, biomarker, genetic, psychosocial, lifestyle etc.) have been consistent throughout both LBC studies and have remained in the protocol for new waves of data collection. At Wave 4 of LBC1921, physical health and medical assessments were expanded upon and new self-report data collected. The primary aim for LBC1921 Wave 5 was to add magnetic resonance imaging (MRI) of the brain with diffusion tensor and magnetization transfer imaging (already available in the LBC1936 sample from Wave 2 onwards) to the cognitive ability data collected at previous waves. A sub-sample of LBC1921 participants (*n* = 42) had previously had structural MRI scans at age 83. A major aim of LBC1936 Waves 3 and 4 was to use longitudinal brain imaging data to examine determinants and consequences of age-related changes in brain structure, and several collaborative opportunities also arose during these waves leading to the collection of new types of data. New measures are described below and refer to data collection in the LBC1936 sample unless otherwise stated. A brief overview of each cohort’s measurement protocol and new variables measured at each wave is given in Table 1 (LBC1921) and Table 2 (LBC1936). Supplementary Table 3 (LBC1921) and Supplementary Table 4 (LBC1936) (available as Supplementary data at *IJE* online) provide, for the first time, comprehensive data grids listing all variables collected throughout both LBC studies and the wave at which they were introduced. These are intended to be useful tools for would-be collaborators with the LBCs.

### New physical, health and biomedical data

At LBC1936 Waves 3 and 4, tests of hearing ability by hear-check screener and self-report (hearing was assessed in LBC1921 Wave 4), and bioelectrical impedance analysis of body composition, were added to the protocol. Analysis of blood biomarkers was extended from standard haematology and biochemistry to include a larger range of markers of inflammation, oxidative stress and lipoproteins. Additions to the LBC1921 protocol at Wave 4 were hand symmetry analysis (hands scanned on digital flatbed scanner) to be used in the measurement of fluctuating asymmetry of the body, and musculoskeletal history, including history of fractures, joint replacement surgery and diagnosis with osteoarthritis.

A brain tissue bank for post-mortem tissue donation was established at Wave 3 of LBC1936 in collaboration with the Medical Research Council (MRC)-funded University of Edinburgh Brain Banks, to enable examination of synaptic integrity and axonal structure and their relation to cognitive and other lifetime data. Novel tissue preparation methods and high-resolution array tomography and electron microscopy imaging techniques will be applied to examine human brain synapses at a high level of morphological and molecular detail. To date, ∼15% of the original LBC1936 sample have given pre-mortem consent. Stages of post-mortem analyses from tissue collection to imaging, and details of the first LBC1936 post-mortem brain tissue analyses, are presented in a protocol paper.^12^

At Wave 4 of LBC1936, blood samples were collected and a bank of cells from 420 participants was formed for the generation of iPSC. The first batch of cells was reprogrammed in 2017 as part of a collaboration with the University of Edinburgh’s Centre for Regenerative Medicine funded by the MRC via the Dementias Platform UK. These have selected 24 participants based on cognitive ageing between childhood and older age, with eight samples each of healthy, average and unhealthy (but not dementia) lifetime cognitive ageing. iPSCs can be reprogrammed to form any tissue type. LBC1936 iPSCs will be harnessed for the formation of brain cells (neurons and glia) to test directly the effects of environmental challenges on brain tissue, which cannot not be done *in vivo*. Stem cells from the LBC1936 participants have the potential to be used in future collaborative projects and experiments examining both pathological and non-pathological ageing and age-related neurodegenerative conditions.

### New genetic and epigenetic data

Genome-wide testing of single nucleotide polymorphisms (SNPs) was completed previously on the LBCs, using the Illumina 610 quad array. New since the previous profile article^4^ is that whole-genome sequencing of over 1300 members of the LBC1921 and LBC1936 was funded by the Biotechnology and Biological Sciences Research Council (BBSRC), and the data are now available to collaborators.

Using the Illumina Infinium HumanMethylation450 BeadChip, DNA methylation data have now been collected from almost every LBC1921 and LBC1936 participant at all waves of testing (except LBC1921 Wave 2), with funding from Wellcome, the University of Edinburgh, the University of Queensland and Age UK.

Cell lines from previously stored LBC1936 blood samples have also been used to examine gene expression in a new transcriptomics project funded by the University of Edinburgh. Using the Illumina HumanHT-12 v4 Expression BeadChip, genome-wide transcriptional coverage has been added to the LBC1936 data at Waves 1 and 3. Thus far, the data have been used to examine age-related changes in gene expression and their association with cognitive, physical and biochemical traits in older adults.^13^

### New self-report and lifestyle data

New questionnaire data have been added to the LBC1936 protocol since 2012. An adapted version of the Pittsburgh Sleep Quality Index^14^ was administered at Waves 3 and 4 to assess participants’ sleep quality and patterns. Self-reported resilience, measured with the Brief Resilience Scale,^15^ was also recorded at these waves (and LBC1921 Wave 4), as were self-reported memory problems. LBC1921 participants previously provided retrospective data on lifetime engagement in physical, leisure and socio-intellectual activities;^16^ the same questionnaire has since been administered to the LBC1936 following Wave 3. The Attitudes to Ageing Questionnaire,^17^ previously completed at Wave 2 of LBC1936, was administered to LBC1921 at Wave 4. LBC1921 participants completed a qualitative life review questionnaire^18^ at Wave 4, close to their 90th birthday. It was designed to contextualize the cohort in time and place and describe their ‘start in life’, with details on their experiences of family and schooling.

### Lifetime geographical data

A ‘life grid’ questionnaire was administered after LBC1936 Wave 3, in which participants provided retrospective accounts of all addresses they had lived at throughout their lifetime, as well as detailing all occupations held (by themselves and their fathers) from first employment to retirement. As part of the ‘Mobility, Mood and Place’ project funded by the Engineering and Physical Sciences Research Council and in collaboration with University of Edinburgh’s Geography Department and the Landscape Architecture Department,^19^ lifetime address data were geo-coded to estimate historical and longitudinal exposure to environmental factors such as urban green space and pollution in the Edinburgh area of Scotland, and will be used to study the influence of these environmental influences on cognitive ageing.

### Objective physical activity data

A collaborative project, ‘Seniors USP’, set up by researchers at Glasgow Caledonian University and funded by the Medical Research Council, was initiated at Wave 4.^20^^,^^21^ The project aimed to understand the relationship between time spent by older adults sitting (sedentary behaviour) and its effect on health, including cognitive function. Self-reported sedentary behaviour patterns were recorded in questionnaires and were supplemented by a sedentary behaviour diary. Most importantly, objective data were collected: ∼7 days of objective data were recorded by electronic activity monitor in a subset of ∼300 LBC1936 participants. An additional sub-sample (*n* = 16), who had worn the activity monitor, provided qualitative accounts of their understanding of sedentary behaviour during semi-structured interview.

### New data linkage

Following completion of dementia ascertainment in the LBC1921,^22^ linkages with morbidity and mortality were extended to work on dementia ascertainment, chronic disease and survival in the LBC1936. Funded by the Alzheimer Scotland Dementia Research Centre, LBC1936 dementia ascertainment is being conducted using various available sources of information, to give an indication of the number of participants potentially affected by the condition as it becomes more prevalent in the younger cohort. This provides the opportunity to study whether there are early signs to predict dementia and to search for risk factors that might be associated with the condition.

### New scientific aims

The newly collected data enhance the wealth of data already collected in the LBC studies. Multiple waves of cognitive ability, psychosocial, well-being, lifestyle, genetics, other ’omics, brain imaging, physical fitness and biomedical data add to the historically recorded and retrospectively recalled life course data, which means that the studies have the potential to address a number of key research questions which go beyond cognitive and brain ageing. For example, the LBCs can also be used to examine the nature and determinants of the ageing-related trajectories of physical and mental health and well-being more broadly. The following is a brief indication of some possible analyses.

The availability of four waves of LBC1936 data and five waves of LBC1921 data will allow us and others to investigate the structure and determinants of age-related cognitive and brain ageing, including latent growth curve and mediation and moderation models. Machine learning methods may be applied in the exploration of cognitive ageing determinants and, combined with forthcoming dementia ascertainment data, could allow us to build predictive models of dementia status. New biomedical and lifestyle data can contribute to an evidence base for identifying modifiable factors that influence ageing, and have the potential to inform interventions to ameliorate the detrimental effects of ageing. Whole-genome sequencing data, probably as part of a consortium, have the potential to further the understanding of the molecular genetic contributions to cognitive decline and other important phenotypes. Longitudinal epigenetic data may be used to explore associations between epigenetic age acceleration and important aspects of ageing such brain and cognitive change, mood and physical and mental health. The LBCs contain assessments of several putative biomarkers of ageing; they can, therefore, be used to test how these relate to each other, and whether they make complementary contributions to the prediction of cognitive, physical and mental health and mortality. It should be stressed, again, that data available have the potential to answer questions far beyond those listed here and emphatically not limited to cognitive ageing; collaboration and data sharing are encouraged. Readers wishing to formulate research ideas are urged to scrutinise Supplementary Tables 3 and 4 where there is a fuller list of variables, including measurement details, that are available from each wave of the LBC1921 and LBC1936 studies.

## What has it found? Key findings and publications

Together, LBC1921 and LBC1936 data have contributed to about 400 peer-reviewed publications. Since the original cohort profile,^4^ the studies have continued to produce new findings in the fields of cognitive ageing and epidemiology. A narrative overview of key cognitive and brain ageing results from almost 20 years of research on the LBC studies is available elsewhere.^23^ Here we give an update on some results published since the 2012 profile, mainly stemming from new data types or resulting from new longitudinal analyses. Results are discussed according to five main areas of research: cognitive ageing, brain imaging, genetics and epigenetics, medical and physical health, and lifestyle. A fuller list of publications, including those published between 2012 and 2017 and not reported here, can be found on the LBC website [www.lothianbirthcohort.ed.ac.uk].

### Cognitive ageing

Given the unusual separation in time between the original intelligence test at age 11 and repeat testing in old age, a central contribution of the LBC studies has been to examine intelligence across most of the lifespan. They have demonstrated that childhood cognitive ability accounts for ∼50% of the variance in cognitive ability in older age,^9^ and that individual differences in intelligence continue to be stable across almost the entire human life span, into the tenth decade.^10^^,^^11^ Continued assessment of cognitive function, through the eighth (LBC1936), ninth and into the 10th decade (LBC1921), means that the studies are well placed to examine the structure of cognitive function within older age.^24^^,^^25^ Evidence from the LBC1936 indicates that a large proportion of variation in age-related declines across a range of cognitive functions is shared: about 50% of the variance in cognitive change on 13 cognitive subtests was accounted for by a factor of general cognitive change.^25^ Furthermore, results indicating a strong association between general cognitive change and change in speed of basic visual information processing^26^ suggest that age-related declines in cognitive ability are related to efficiency of perceptual discrimination, as suggested in the processing speed theory of cognitive ageing.^27^

Repeated collection of cognitive and other phenotypic data has also enabled the cohorts to examine putative determinants of cognitive ageing. Many of the variables tested, including childhood IQ, have been found to relate only with later life ability levels,^25^ and not with subsequent trajectories of cognitive decline. In multivariate analyses, possession of the *APOE* e4 allele is one of the few robust independent predictors of relatively greater older-age cognitive decline.^25^ There is some evidence that physical fitness may be protective against decline,^16^^,^^25^ though physical and cognitive processes in general may be influenced by different ageing processes, because evidence for cognitive and physical functions declining in tandem is minimal.^28^

### Brain imaging

Wave 3 of LBC1936 extended current understanding of the ageing brain through longitudinal examination of structural brain imaging data. From Wave 2, the major hypotheses of the LBC1936 study were based on a theory of brain white matter disconnection as one of the contributors to individual differences in cognitive ageing. Longitudinal analyses showing that microstructural integrity of the white matter tracts declines in concert with fluid intelligence in older age provided some of the strongest evidence for this theory to date.^29^ Other structural brain features have also been found to be neuro-anatomical contributors to cognitive ageing, including cortical thickness,^29^ volumes of healthy grey and white matter and indicators of small vessel disease.^30–33^ LBC1936 data have previously shown that the associations between intelligence and brain structure are present across much of the life course.^30^ New, within-older-age longitudinal results show that cognitive and brain structural changes are also related throughout older age. Individuals who score higher on tests of memory and information processing speed at age 73 show less subsequent change in total brain and white matter volume, and brain volume atrophy due to loss of grey and white matter and progression of white matter hyperintensities is modestly associated with declines in memory, speed and fluid intelligence between ages 73 and 76 years.^31^

As with cognitive change within older age, predictors of brain structural changes from age 73 to age 76 have remained elusive, and the effect sizes for significant associations have been small. In multivariate studies, few predictors of initial brain structure are associated with brain structural change, *APOE* variation and physical fitness differences again being the most robust associates.^34^ Separate studies using LBC1936 data have indicated: that those adhering to a Mediterranean diet have less total brain atrophy between ages 73 and 76;^35^ and that vascular risk factors, including poorer glycaemic control (in *APOE* e4 carriers),^36^ lower HDL cholesterol^37^ and smoking,^37^^,^^38^ are possibly detrimental to brain health, i.e. they are associated with cortical thinning and white matter hyperintensity progression. Development of a new biomarker of ‘brain age’ has shown that brain health is important not only for cognitive function, but might also be predictive of physical health and survival.^39^

### Genetics and epigenetics

Epigenetic (DNA methylation) data have been an important addition to the LBC cohorts’ protocols. Variation in epigenetic CpG markers alter gene expression but not the underlying DNA sequence, and are associated with health outcomes independently of genetic profiles.^40^ DNA methylation is susceptible to ageing-related changes. DNA methylation data from both cohorts, used to calculate ‘biological age’, showed that disparities between ‘biological age’ and chronological age (epigenetic age acceleration, or a faster-running ‘epigenetic clock’) are related to general bodily fitness and cognitive ability^41^ and are predictive of mortality.^42^ LBC data have contributed to epigenome-wide association studies which have found associations between methylation levels and markers of chronic low-grade inflammation^43^ and BMI,^44^ and between smoking-related DNA methylation differences and genes associated with smoking-related illnesses, such as pulmonary and cardiovascular disease, osteoporosis, rheumatoid arthritis and cancer.^45^

In addition to new epigenetic variables being measured, existing genetic data have been used to form new measurement types. Polygenic risk scores are one example of this. Increased polygenic risk for schizophrenia in individuals without the disorder was associated with lower fluid-type general cognitive ability at age 70, and greater cognitive decline between age 11 and 70 years.^46^ LBC data have also contributed to polygenic risk studies indicating that there may be overlap in genetic variants associated with poorer cognitive ability and coronary artery disease^47^ and ischaemic stroke,^48^ but not with Alzheimer’s Disease (AD)^49^ or diabetes.^50^ The LBC1936 dataset was used to show that, among those individuals with a higher age-11 IQ score, there was a significantly weaker association between the polygenic risk score for type 2 diabetes and HbA1c at age 70 than in those with lower IQ scores.^51^ Therefore, childhood IQ acts as a moderator of the genetic tendency to type 2 diabetes.

### Medical and physical health

Several biomedical and physical health factors have been studied in relation to cognitive ageing between childhood and older age. For future studies, post-mortem examination of brain tissue has extended cognitive and brain phenotyping to the level of the synapse. Presently, the availability of multiple waves of cognitive data from both cohorts has enabled examination of the influence of medical and physical factors on cognitive change within older age.

Data from LBC1936 have shown that vascular stiffening, as indexed by increased pulsatility and resistivity of the internal carotid artery, but not carotid luminal narrowing, is associated with poorer performance on tests of processing speed and visuo-spatial function and decreased crystallized intelligence between the ages of 70 and 76.^52^ However, quantitative microvascular differences assessed from retinal fundus images have shown few associations with a range of cognitive abilities, providing limited support for their use as a marker of non-pathological cognitive ageing.^53^ In LBC1936, lower levels of crystallized, visuopatial, memory and processing speed ability have also been associated with greater physical frailty in older age, and decline in processing speed between the ages of 70 and 76 is also related to increased risk of physical frailty onset.^54^

Data from LBC1921 participants have contributed to a study of dementia in the SMS1932 cohort from which they originated. Results indicated that lower IQ at age 11 is related to increased risk of dementia diagnosis between age 65 and 92, an effect which is stronger for females than for males.^55^ Probable dementia has been ascertained in ∼20% of LBC1921; positive *APOE* e4 carrier status and greater participation in physical activity across the life course were found to be risk factors for dementia, whereas a history of hypertension and increased height may reduce the risk of dementia diagnosis between ages 79 and 95.^22^

### Lifestyle

Data on current and retrospective levels of engagement in leisure, socio-intellectual and physical activity from the LBC1921 study suggest that the importance of different types of activities in relation to cognitive ageing may vary across the life course.^16^ A study which aimed to identify ecological determinants of sedentary behaviour, and included LBC1936 data, found that being in a carer role was related to lower levels of sedentary behaviour, and increased sedentary behaviour was related to a fear of crime and perceived absence of neighbourhood services.^20^ Greater social disadvantage measured using a range of socioeconomic position measures has also been associated with increased time spent sedentary among retired individuals.^21^ Examination of the impact of home and neighbourhood environment on health and cognitive function has expanded from indices of deprivation to include the influence of urban space exposure, particularly access to green space, with greater access to neighbourhood public parks, particularly in childhood, shown to relate to lower rates of cognitive decline in later life.^56^

## What are the main strengths and weaknesses?

An unusual strength of both cohorts is the possession of a valid intelligence test score from age 11 years in people who are in older age, and who have taken the same intelligence test again throughout older age along with a range of other cognitive and many other tests and questionnaires and diverse assessments. This means that cognitive changes from childhood across most of the lifespan can be described and explained, and allows us to test for possible confounding or reverse causation between lifetime intelligence and putative determinants of cognitive ageing, whereby apparent determinants of individual differences in cognitive ageing (such as lifestyle factors) are themselves predicted by differences in intelligence measured in childhood. Key examples of confounding or reverse causation in LBC1936 include diet,^57^ diabetes^58^ and participation in intellectual activities,^59^ with associations between each of these variables and older-age cognitive ability being confounded by previous IQ. Combined with structural brain imaging in both cohorts, the studies have the ability to examine the biological foundations of cognitive function throughout most of older age. It is also a strength that both LBC studies are narrow age cohorts, which means that the variables measured reflect differential ageing and not chronological age, which can otherwise swamp the variance in assessments and inflate effect sizes. With there being two LBC cohorts, born 15 years apart and tested on the same childhood intelligence test and on many similar variables at the same older age (79 years), it is possible to perform some tests of cohort differences. This will be done by matching the LBC1921 and LBC1936 on key background variables and then testing for differences in outcomes of interest. The prospective data collection from a variety of sources on a broad range of putative determinants and outcomes, many of which are repeated at multiple waves, means that both cohorts are rich sources of data.

Weaknesses of the LBCs include the limited sample sizes of the two cohorts, their being somewhat restricted in range with respect to childhood intelligence and childhood and adult socioeconomic status, and there being several decades without data collection between participants sitting the MHT at age 11 and the LBC studies being initiated when they were aged 70 (LBC1936) and 79 (LBC1921). The mean (SD) Moray House Test score at age 11 was: 46.4 (12.1) for the LBC1921 compared with 34.5 (15.5) for the whole of Scotland; and 49.0 (11.8) for the LBC1936 compared with 36.7 (16.1) for the whole of Scotland, and a mean of 40.3 for the Edinburgh region. However, the reports on the samples are able—because the whole populations were tested at age 11—to estimate accurately by how much these factors might attenuate effect sizes. Longitudinal attrition has resulted in some sampling bias, such that those who continue to participate in testing are more cognitively and physically able to do so, though this can be addressed using up-to-date statistical techniques. Both cohorts are also limited by being entirely composed of Caucasian participants. 

## Can I get hold of the data? Where can I find out more?

The LBCs’ study data have been the subject of many internal (within the University of Edinburgh) and external collaborations, which are encouraged. Those who have interests in outcomes other than cognitive domains are particularly encouraged to collaborate. The current procedure for those who wish to work with the data is initially to e-mail the Lothian Birth Cohorts Director Professor Ian Deary to ask for an ‘LBC Data Request Form’. Comprehensive data grids listing all variables collected throughout both LBC studies and the wave at which they were introduced are provided in Supplementary Tables 3 and 4, and both LBC studies have clear data dictionaries which help researchers to discern whether the variables they wish to use are present; these provide a simple short title for each variable, alongside a longer, common-sense description/provenance of each variable. The process is facilitated by a full-time LBC database manager. Such proposals, when approved, are conducted in collaboration with appropriate members of the LBC study team.

**Lothian Birth Cohorts (LBCs) in a nutshell**

- The LBCs originated as follow-ups to the Scottish Mental Surveys of 1932 (SMS1932) and 1947 (SMS1947) which tested childhood intelligence in Scotland.
- The Lothian Birth Cohorts of 1921 (LBC1921: *N* = 550, beginning at age 79) and 1936 (LBC1936: *N* = 1091, beginning at age 70) began in 1999 and 2004, respectively, principally to study determinants of non-pathological cognitive ageing.
- Both cohorts have been followed up at ∼3-yearly intervals. New waves of data have been collected since the previous LBCs’ profile. The most recent waves were completed in 2013 (LBC1921 Wave 5: *n* = 59, age 92) and 2017 (LBC1936 Wave 4: *n* = 550, age 79).
- A broad range of cognitive, psychosocial, brain imaging, medical, physical, genetic, lifestyle and other variables have been collected longitudinally.
- New measures since the previous LBCs’ cohort profile include: whole-genome sequencing, longitudinal DNA methylation, longitudinal gene expression, lipidomics, sedentary patterns analysis, inducible pluripotent stem cells (iPSC), historical environmental exposures, post-mortem brain tissue, dementia ascertainment (LBC1936) and several new biomarkers and questionnaire assessments.
- The cohorts have several existing collaborations. For enquiries regarding new collaborative opportunities and data sharing, please contact the LBCs’ study Director Ian Deary [i.deary@ed.ac.uk].

## Funding

LBC1921 funding has been received from the UK's Biotechnology and Biological Sciences Research Council (BBSRC) (15/SAG09977, wave 1), a Royal Society-Wolfson Research Merit Award to IJD (wave 2), the Chief Scientist Office (CSO) of the Scottish Government's Health Directorates (CZG/3/2/79, post-wave 1 questionnaire study; CZB/4/505, wave 3; ETM/55, wave 4), and the UK's Medical Research Council (MRC) Centenary Early Career Award to Dr Tom Booth (wave 5). Funding for LBC1936 has been received from Research Into Ageing (Programme grant 251; wave 1), and Age UK (Disconnected Mind Programme grant) and the UK's Medical Research Council (G0701120, wave 2; G1001245, wave 3; MR/M013111/1, wave 4). The Alzheimer Scotland Dementia Research Centre funded LBC1936 dementia ascertainment. BBSRC funded whole-genome sequencing of both cohorts. Wellcome, the University of Edinburgh, the University of Queensland, and Age UK funded DNA methylation analysis in both cohorts. Ian Deary is supported by the University of Edinburgh Centre for Cognitive Ageing and Cognitive Epidemiology which is funded by the Medical Research Council and the Biotechnology and Biological Sciences Research Council (Grant No. MR/K026992/1).

**Conflict of interest:** None declared. 

## Supplementary Material

Supplementary Data
