# Supplementary material for: Cohort Profile Update: The Lothian Birth Cohorts of 1921 and 1936
Source: Int J Epidemiol. 2018 Mar 12;47(4):1042–1042r. doi: 10.1093/ije/dyy022 (PMC6124629; doi:10.1093/ije/dyy022)
Supplement: Supplementary Data [file dyy022_supplementary_tables.doc]

Supplementary Table 1. Characteristics of Completers by wave in the Lothian Birth Cohort 1921 (N = 59)

| Variable | Wave 1 | Wave 2 | Wave 3 | Wave 4 | Wave 5 |
| --- | --- | --- | --- | --- | --- |
| MMSE | 28.8 (1.35) | 29.0 (1.03) | 28.6 (1.35) | 28.0 (1.47) | 27.2 (2.36) |
| CVD (yes)^a^ | 23.8% | 13.6% | 25.4% | 44.1% | 50.8% |
| Grip strength^b^ | 28.5 (9.60) | 26.6 (10.2) | 22.9 (9.06) | 21.0 (7.66) | 17.9 (9.11) |
| FEV_1_ | 2.06 (0.52) | - | 1.84 (0.52) | 1.71 (0.52) | 1.53 (0.54) |

*Note*. N = 59. Values given are mean and standard deviation, except for sex and CVD, for Completers at each wave. MMSE = Mini-Mental State Examination. CVD = cardiovascular disease. FEV_1_ = forced expiratory volume from the lungs in one second.

^a^ CVD includes unsure and angina only at Wave 1

^b^ Grip strength reported as best of three from the right hand.

Supplementary Table 2. Characteristics of Completers by wave in the Lothian Birth Cohort 1936 (N=550).

| Variable | Wave 1 | Wave 2 | Wave 3 | Wave 4 |
| --- | --- | --- | --- | --- |
| MMSE | 28.9 (1.30) | 28.9 (1.25) | 28.8 (1.53) | 28.5 (2.16) |
| CVD (yes) | 22.7% | 28.2% | 33.6% | 37.1% |
| Grip strength^a^ | 29.5 (10.2) | 28.9 (9.40) | 27.8 (9.84) | 26.1 (9.43) |
| FEV_1_ | 2.46 (0.68) | 2.35 (0.69) | 2.15 (0.64) | 2.11 (0.64) |

*Note*. Values given are mean and standard deviation, except for sex and CVD, for Completers at each wave. MMSE = Mini-Mental State Examination. CVD = cardiovascular disease. FEV_1_ = forced expiratory volume from the lungs in one second.

^a^ Grip strength reported as best of three from the right hand.

Supplementary Table 3. Data grid of all variables measured and waves of collection in the Lothian Birth Cohort 1921 study

Table key:

= data available at time of previous cohorts profile publication

= new wave of data, repeat collection of previous variable

= new variable not previously collected.

| Variable | Historical | Wave 1 | Wave 2 | Wave 3 | Wave 4 | Wave 5 |
| --- | --- | --- | --- | --- | --- | --- |
| Cognitive ability |  |  |  |  |  |  |
| Moray House Test No. 12 (MHT)^1^ |  |  |  |  |  |  |
| Mini-Mental State Examination (MMSE)^2^ |  |  |  |  |  |  |
| Verbal Fluency^3^ |  |  |  |  |  |  |
| Raven’s Progressive Matrice^4^ |  |  |  |  |  |  |
| Wechsler Adult Intelligence Scale-Revised - Digit Symbol Coding^5^ |  |  |  |  |  |  |
| Wechsler Adult Intelligence Scale III^UK^ - Letter-Number Sequencing^6^ |  |  |  |  |  |  |
| Wechsler Memory Scale-Revised- Logical Memory I & II^7^ |  |  |  |  |  |  |
| National Adult Reading Test (NART)^8^ |  |  |  |  |  |  |
| Wechsler Test of Adult Reading (WTAR)^9^ |  |  |  |  |  |  |
| Spot the Word^10,11^ |  |  |  |  |  |  |
| Simple and Choice reaction time^12^ |  |  |  |  |  |  |
| Inspection Time^13^ |  |  |  |  |  |  |
| Social and demographic data |  |  |  |  |  |  |
| Age (in days) |  |  |  |  |  |  |
| Current address |  |  |  |  |  |  |
| Living arrangement (living alone/not alone) |  |  |  |  |  |  |
| Residence type (home owned/rented/residential/nursing/hospital/other) |  |  |  |  |  |  |
| Marital status (single/married/cohabiting/divorced/widowed/other) |  |  |  |  |  |  |
| Home help (times per week) |  |  |  |  |  |  |
| Place of Birth |  |  |  |  |  |  |
| Childhood address and overcrowding/deprivation details  (number of rooms and occupants in home, number sharing toilet, toilet indoor/outdoor) |  |  |  |  |  |  |
| Schools attended |  |  |  |  |  |  |
| Education (years in full-time education) |  |  |  |  |  |  |
| Main occupation of self (and spouse if female) |  |  |  |  |  |  |
| Socio-economic status (SES) coding^14^ |  |  |  |  |  |  |
| Questionnaires |  |  |  |  |  |  |
| Your family (parent’s years in full-time education & main occupation, parent’s & grandparent’s place of birth) |  |  |  |  |  |  |
| Your household, transport, local amenities, loneliness, social support^15^, feelings about yourself^16^ and your life^17^ |  |  |  |  |  |  |
| Significant Others Scale (adapted from the Significant Others (SOS) Scale)^18^ |  |  |  |  |  |  |
| Your children (sex, education and occupation) |  |  |  |  |  |  |
| Employment (main occupation, years in employment, retirement), job situations^19^, job complexity^20^ and mental work demands^21,22^ |  |  |  |  |  |  |
| Retrospective participation in household work^23,24^ |  |  |  |  |  |  |
| Retrospective perceived social support^25-28^ |  |  |  |  |  |  |
| Retrospective lifetime activity participation (sport and exercise, intellectual and social activities)^29-34^ |  |  |  |  |  |  |
| Religious activity(religious group attendance)^35^, Religious Involvement Inventory^36^ , Spiritual Wellbeing Scale^37^ |  |  |  |  |  |  |
| Neighbourhood questions (adapted from the Neighbourhood Cohesion Scale)^38^ |  |  |  |  |  |  |
| Instrumental Activities of Daily Living Scale^39^ |  |  |  |  |  |  |
| Activity lifestyle^40,41^ |  |  |  |  |  |  |
| Typical Intellectual Engagement^42,43^ |  |  |  |  |  |  |
| Hospital Anxiety and Depression Scale (HADS)^44^ |  |  |  |  |  |  |
| World Health Organisation Quality of Life (WHOQOL-BREF)^45^ |  |  |  |  |  |  |
| Satisfaction With Life Scale (SWLS)^46^ |  |  |  |  |  |  |
| International Personality Item Pool Five Factor Personality (IPIP)^47,48^ |  |  |  |  |  |  |
| Warwick-Edinburgh Mental Wellbeing Scale (WEMWBS)^49^ |  |  |  |  |  |  |
| LOT-R (Life Orientation Test-Revised)^50^ |  |  |  |  |  |  |
| Sense of Coherence (adapted from the Sense of Coherence Scale)^51^ |  |  |  |  |  |  |
| The Brief Resilience Scale^52^ |  |  |  |  |  |  |
| Attitudes to Ageing Questionnaire (AAQ)^53^ |  |  |  |  |  |  |
| Life review questionnaire^54^ |  |  |  |  |  |  |
| Physical and Medical |  |  |  |  |  |  |
| Self-rated health^55^ |  |  |  |  |  |  |
| Townsend Disability Scale^56,57^ |  |  |  |  |  |  |
| Smoking history (current/ex/never, age at starting (Wave 1) & stopping, number cigarettes smoked per day) |  |  |  |  |  |  |
| Alcohol consumption (alcohol type and units per week) |  |  |  |  |  |  |
| Self-reported history of disease (Waves 1-5: cardiovascular disease, cerebrovascular disease, cancer, hypertension, diabetes, thyroid disorder, dementia, other vascular disease, other disease; Wave 2-5: eye disease; Waves 4&: hypercholesterolemia, arthritis, allergies) |  |  |  |  |  |  |
| Family history of disease (Waves 4&5: cardiovascular disease, cerebrovascular disease, dementia, Parkinson’s disease) |  |  |  |  |  |  |
| Current medications |  |  |  |  |  |  |
| Height (cm) |  |  |  |  |  |  |
| Weight (kg) |  |  |  |  |  |  |
| BMI |  |  |  |  |  |  |
| Demi-Span (cm) |  |  |  |  |  |  |
| Head circumference (cm) |  |  |  |  |  |  |
| Facial symmetry analysis |  |  |  |  |  |  |
| Fluctuating asymmetry |  |  |  |  |  |  |
| Hand scan for asymmetry analysis |  |  |  |  |  |  |
| Dentition (number of teeth/year lost) |  |  |  |  |  |  |
| Musculoskeletal history |  |  |  |  |  |  |
| Visual acuity (Waves 1,3: at 6 metres measured by Snellen chart (corrected and uncorrected); Wave 2:Log MAR D & N, contrast sensitivity) |  |  |  |  |  |  |
| Hearing (self-reported^58^ and measured with HearCheck screener at 1000Hz and 3000Hz) |  |  |  |  |  |  |
| Systolic and diastolic blood pressure (sitting and standing) |  |  |  |  |  |  |
| Electrocardiogram (ECG) |  |  |  |  |  |  |
| Ankle-brachial pressure index (ABPI) |  |  |  |  |  |  |
| Lung Function (peak expiratory flow rate, forced expiratory volume in 1 second, forced vital capacity) |  |  |  |  |  |  |
| Grip strength (Wave 1: dominant hand (kg); Waves 2-5: left and right hand (kg)) |  |  |  |  |  |  |
| Time to walk 6 metres (in seconds) |  |  |  |  |  |  |
| Chair stand test |  |  |  |  |  |  |
| Biochemistry and haematology analysis |  |  |  |  |  |  |
| Haemoglobin |  |  |  |  |  |  |
| Red cell count |  |  |  |  |  |  |
| Haematocrit |  |  |  |  |  |  |
| Mean cell volume |  |  |  |  |  |  |
| White cell count |  |  |  |  |  |  |
| Neutrophil count |  |  |  |  |  |  |
| Lymphocyte count |  |  |  |  |  |  |
| Monocyte count |  |  |  |  |  |  |
| Eosinophil count |  |  |  |  |  |  |
| Basophil count |  |  |  |  |  |  |
| Platelet count |  |  |  |  |  |  |
| Prothrombin time (PT) |  |  |  |  |  |  |
| PT ratio |  |  |  |  |  |  |
| Activated partial thromboplastin time (APTT) |  |  |  |  |  |  |
| APTT ratio |  |  |  |  |  |  |
| Fibrinogen |  |  |  |  |  |  |
| D-Dimer |  |  |  |  |  |  |
| D-Dimer vidas |  |  |  |  |  |  |
| Vitamin B12 |  |  |  |  |  |  |
| Red cell folate |  |  |  |  |  |  |
| Serum folate |  |  |  |  |  |  |
| Urea |  |  |  |  |  |  |
| Creatinine |  |  |  |  |  |  |
| Estimated glomerular filtration rate (eGFR) |  |  |  |  |  |  |
| Sodium |  |  |  |  |  |  |
| Potassium |  |  |  |  |  |  |
| Albumin |  |  |  |  |  |  |
| Triglyceride |  |  |  |  |  |  |
| Total serum cholesterol |  |  |  |  |  |  |
| Glycated haemoglobin (HbA1c) |  |  |  |  |  |  |
| C-reactive protein (CRP) |  |  |  |  |  |  |
| Thyroid stimulating hormone (TSH) |  |  |  |  |  |  |
| Thyroxine (Wave 1: Total T3, Free Thyroxine; Wave 3: Total T3, Free T4) |  |  |  |  |  |  |
| Inflammation markers |  |  |  |  |  |  |
| Von Willbrand Factor (VWF) |  |  |  |  |  |  |
| Interleukin 6 (IL-6) |  |  |  |  |  |  |
| Tumor necrosis factor alpha (TNFa) |  |  |  |  |  |  |
| Oxidative Stress |  |  |  |  |  |  |
| Trolox equivalent antioxidant capacity (TEAC) |  |  |  |  |  |  |
| DNA damage |  |  |  |  |  |  |
| Antioxidant vitamins |  |  |  |  |  |  |
| Saliva analysis |  |  |  |  |  |  |
| DNA (if unable to obtain blood sample only) |  |  |  |  |  |  |
| Genetic analysis |  |  |  |  |  |  |
| Apolipoprotein E (APOE) genoptyping to determine APOE E4 allele status^59^ |  |  |  |  |  |  |
| Genome-wide genotyping |  |  |  |  |  |  |
| Whole genome sequencing |  |  |  |  |  |  |
| Telomere length |  |  |  |  |  |  |
| DNA methylation^60,61^ |  |  |  |  |  |  |
| Brain magnetic resonance imaging (MRI) |  |  |  |  |  |  |
| Structural brain MRI measures (various; sub-sample n = 42) |  |  |  |  |  |  |
| Diffusion tensor (DT-MRI) derived variables of long-range tracts |  |  |  |  |  |  |
| White matter hyperintensity volume |  |  |  |  |  |  |
| Stroke lesion volume |  |  |  |  |  |  |
| Intracranial volume |  |  |  |  |  |  |
| Hippocampal volume |  |  |  |  |  |  |
| Total brain tissue volume |  |  |  |  |  |  |
| Normal appearing white matter volume |  |  |  |  |  |  |
| Grey matter volume |  |  |  |  |  |  |
| Separate and total ventricular volume |  |  |  |  |  |  |
| Visual atrophy and lesion rating scales |  |  |  |  |  |  |
| Desikan-Killiany Atlast Parcellation (FreeSurfer) – volume and surface area^62^ |  |  |  |  |  |  |
| Neuroradiological report |  |  |  |  |  |  |
| Retinal imaging |  |  |  |  |  |  |
| Semi-automated quantitative measures of retinal microvascular widths and branching geometry^63^ |  |  |  |  |  |  |
| Linkages |  |  |  |  |  |  |
| Date and cause of death |  |  |  |  |  |  |
| Morbidity |  |  |  |  |  |  |
| Dementia diagnosis |  |  |  |  |  |  |

**Supplementary References (for Supplementary Table 3)**

1. Scottish Council for Research in Education. *The intelligence of Scottish Children*. London, England: University of London Press, 1933.
2. Folstein MF, Folstein SE, McHugh PR. Mini-Mental State: A practical method for grading the cognitive state of patients for the clinician. *J Psychiat Res* 1975; 12:189-198.
3. Lezak M. *Neuropsychological testing*. Oxford, England: Oxford University Press, 1995.
4. Raven JC, Court JH, Raven J. *Manual for Raven’s progressive matrices and vocabulary scales.* London, England: H. K. Lewis, 1977.
5. Wechsler D. *Wechsler Adult Intelligence Scale-Revised.*  New York, NY: Psychological Corp, 1981.
6. Wechsler D. *WAIS-IIIUK administration and scoring manual*. London, UK: Psychological Corporation, 1998.
7. Wechsler D. *Wechsler Memory Scale-Revised*. New York, NY: Psychological Corp, 1987.
8. Nelson HE, Willison JR. *National Adult Reading Test (NART) Test Manual (Part II).* Windsor, UK: NFER-Nelson, 1991.
9. Holdnack JA. *WTAR: Wechsler Test of Adult Reading manual.* San Antonio, TX: Psychological Corporation, 2001.
10. Baddeley A, Emslie H, Nimmo-Smith I. The Spot-the-Word test: A robust estimate of verbal intelligence based on lexical decision. *Br J Clin Psychol* 1993, 32:55-65.
11. Baddeley A, Crawford A. *Spot the Word, Second Edition*. Oxford, England: Pearson Assessment, 2012.
12. Deary IJ, Der G, Ford G. Reaction times and intelligence differences: a population-based cohort study. *Intelligence* 2001; 29:389-399.
13. Deary IJ, Simonotto E, Meyer M *et al*. The functional anatomy of inspection time: an event-related fMRI study. *Neuroimage* 2004; 22:1466-1479.
14. General Register Office. *Census 1951: classification of occupations*. London, UK: Her Majesty’s Stationary Office, 1956.
15. Benzeval M *et al*. Cohort profile: West of Scotland Twenty-07 Study: Health in the Community. *Int J Epidemiol* 2009; 38:1215-1223.
16. Rosenberg, M. *Society and the adolescent self-image*. Princeton, NJ: Princeton University Press, 1965.
17. Pearlin LI, Schooler C. The Structure of Coping. *Journal of Health and Social Behaviour* 1978; 19:2-21.
18. Power MJ, Champion LA, Aris SJ. The development of a measure of social support: the Significant Others (SOS) Scale. *Br J Clin Psychol* 1988; 27:349-358.
19. Karasek R, Brisson C, Kawakami N, Houtman I, Bongers P, Amick B. The Job Content Questionnaire (JCQ): an instrument for internationally comparative assessments of psychosocial job characteristics. *J Occup Health Psychol*; 1998;3:322-55.
20. Vaananen A. (2004). Job Complexity. Ref Type: Personal Communication.
21. De Zwart B, Broerson J, Van der Beek A, Frings-Dresen M, Van Dijk F. Occupational classification according to work demands: an evaluation study. *Int J Occup Med Environ Health* 1997; 10, 283-295.
22. Bosma H, van Boxtel MPJ, Ponds RWHM, Houx PJ, Burdorf A, Jolles J. Mental work demands protect against cognitive impairment: MAAS Prospective Cohort Study. *Exp Aging Res* 2003a; 29, 33-45.
23. Frandin K, Mellstrom D, Sundh V, Grimby G. A life span perspective on patterns of physical activity and functional performance at the age of 76. *Gerontology* 1995; 41, 109-120.
24. Friedenreich C, Courneya, KS, Bryant HE. The Lifetime Total Physical Activity Questionnaire: development and reliability. *Med Sci Sports and Exerc* 1998; 266-274.
25. Seeman TE, Berkman LF. Structural characteristics of social networks and their relationship with social support in the elderly: who provides support. *Soc Sci Med* 1988; 26, 737-749.
26. Bassuk SS, Glass TA, Berkman LF. Social disengagement and incident cognitive decline in community-dwelling elderly persons. *Ann Intern Med* 1999; 131, 165-173.
27. Sherbourne CD, Stewart AL. The MOS social support survey. *Soc Sci Med* 1991; 32, 705-714.
28. Sarason IG, Sarason BR, Shearin EN, Pierce GR. A brief measure of social support: practical and theoretical implications. *J Socl Pers Relat* 1987; 4, 497-5.
29. Hirvensalo M, Lampinen P, Rantanen T. Physical exercise in old age: an eight-year follow-up study on involvement, motives, and obstacles among persons age 65-84. *J Aging Phys Act* 1998; 6, 157-168.
30. Hultsch DF, Hertzog C, Small BJ, Dixon RA. Use it or lose it: engaged lifestyle as a buffer of cognitive decline in aging? *Psychol Aging* 1999; 14, 245-263.
31. Glass TA, de Leon CM, Marottoli RA, Berkman LF. Population based study of social and productive activities and as predictors of survival among elderly Americans. *BMJ* 1999; 319, 478-483.
32. Richards M, Hardy R, Wadsworth MEJ. Does active leisure protect cognition? Evidence from a national birth cohort. *Soc Sci Med* 2003; 56, 785-792.
33. Wilson RS, Barnes LL, Bennett DA. Assessment of lifetime participation in cognitively stimulating activities. *J Clin Exp Neuropsychol* 2003; 25, 634-642.
34. Wilson RS, Beckett LA, Barnes LL, Schneider JA, Bach J, Evans DA *et al*. Individual differences in rates of change in cognitive abilities of older persons. *Psych Aging* 2002; 17, 179-193.
35. Gow AJ, Watson R, Whiteman M, Deary IJ. A stairway to heaven? Structure of religious involvement inventory and spiritual well-being scale*. J Relig Health* 2011; 50:5-19.
36. Hilty DM, Morgan RL. Construct validation for the religious involvement inventory: replication. *J Sci Stud Relig* 1985;24:75-86.
37. Paloutzian RF, Ellison CW. *Loneliness, spiritual well-being and the quality of life.* In Peplau LA & Perlman D (eds.). Loneliness: a sourcebook of current theory, research and therapy (pp. 224-237). New York, NY: Wiley. (1982).
38. Buckner JC. The development of an instrument to measure neighbourhood cohesion. *Am J Community Psychol* 1988; 16:771-791.
39. Myers AM. The clinical swiss army knife: Empirical evidence on the validity of IADL functional status measures*. Med Care* 1992; 30:MS96-MS111.
40. Gow AJ, Pattie A, Deary IJ. Lifecourse activity participation from early, mid, and later adulthood as determinants of cognitive aging: the Lothian Birth Cohort 1921*. J Gerontol B Psychol Sci Soc Sci* 2017; 72:25-37.
41. Glass TA, de Leon CM, Marottoli RA, Berkman LF. Population based study of social and productive activities and as predictors of survival among elderly Americans. *BMJ* 1999; 319:478-483.
42. Gow AJ, Whiteman MC, Pattie A, Deary IJ. The personality-intelligence interface: insights from an ageing cohort. *Personality and individual differences* 2005; 39:751-761.
43. Goff M, Ackerman PL. Personality-intelligence relations: assessment of typical intellectual engagement*. Journal of Educational Psychology* 1992; 84:537-552.
44. Zigmond AS, Snaith RP. The Hospital Anxiety and Depression Scale. *Acta Psyschiatr Scand* 1983; 67:361-370.
45. Group WHOQOL: Development of the World Health Organization WHOQOL-BREF Quality of Life Assessment. *Psychol Med* 1998, 28:551–558.
46. Diener E, Emmons RA, Larsen RJ, Griffin S. The Satisfaction With Life Scale. *J Pers Assess* 1985; 49:71-75.
47. Goldberg LR. *A broad-bandwidth public domain, personality inventory measuring the lower-level facets of several five-factor models*. In I. Mervielde, I. J. Deary, F. de Fruyt, & F. Ostendorf (Eds.). Personality psychology in Europe (Vol. 7, pp. 7–28). Tilburg: Tilburg University Press.
48. Website title: [http://ipip.ori.org/].
49. Tennant R, Hiller L, Fishwick R, Platt S, Joseph S, *et al*. The Warwick- Edinburgh Mental Well-being Scale (WEMWBS): development and UK validation. *Health Qual Life Outcomes* 2007; 5:63
50. Scheier MF, Carver CS, Bridges MW. Distinguishing optimism from neuroticism (and trait anxiety, self-mastery, self-esteem): a re-evaluation of the Life Orientation Test. *J Pers Soc Psychol* 1994; 67:1063–1078.
51. Lundberg O, Nystrom Peck M. A simplified way of measuring sense of coherence: experiences from a population survey in Sweden. *Eur J Public Health* 1995; 5: 56-59.
52. Smith BW, Dalen J, Wiggins K, Tooley E, Christopher P, Bernard J. The Brief Resilience Scale: assessing the ability to bounce back. *Int J Behav Med* 2008; 15:194-200.
53. Laidlaw K, Power MJ, Schmidt S. The Attitudes to Ageing Questionnaire (AAG): development and psychometric properties. *Int J Geriatr Psychiatry* 2007; 22:367-379.
54. Lapsley H, Pattie A, Starr JM, Deary IJ. Life review in advanced age: qualitative research on the ‘start in life’ of 90-year-olds in the Lothian Birth Cohort 1921. *BMC Geriatr* 2016;16:74
55. Ware JE, Sherbourne CD. The MOS 36-Item Short-Form Health Survey (SF-36): 1. Conceptual framework and item selection. *Med Care* 1992; 30: 473-483.
56. Bond J, Carstairs V. *Services for the elderly: a survey if the characteristics and needs of a population of 5,000,000 old people (Scottish Home and Health Studies No. 42)* Edinburgh, Scotland: Scottish Home and Health Department, 1982.
57. Townsend P. *Poverty in the United Kingdom*. Hardmonsworth, England: Pelican, 1979.
58. Akeroyd M. Hearing questionnaire adapted from a postal survey by Stuart Gatehouse, IHR Scottish Section 2004.
59. Wenham PR, Price, WH, Blandell G. Apolipoprotein E genotyping by one-stage PCR. *Lancet* 1991; 337:1158-1159.
60. Hannum G, Guinney J, Zhao L *et al.* Genome-wide methylation profiles reveal quantitative views of human aging rates. *Mol Cell* 2013;49:359-67.
61. Horvath S. DNA methylation age of human tissues and cell types. *Genome Biol* 2013; 14:R115.
62. Desikan RS, Segonne F, Fischl B et al. An automated labelling system for subdividing the human cerebral cortex on MRI scans into gyral based regions of interest. NeuroImage 2006; 31:968-980.
63. Patton N, Pattie A, MacGillivray T *et al*. The Association between Retinal Vascular Network Geometry and Cognitive Ability in an Elderly Population. *Invest Ophthalmol Vis Sci* 2007, 48, 1995-2000.

Supplementary Table 4. Data grid of all variables measured and waves of collection in the Lothian Birth Cohort 1936 study

Table key

Data available:

= data available at previous cohort profile publication (2012)

= new data wave, repeat of previous variable

= new variable, not previously collected

Forthcoming data:

= planned for Wave 5, repeat of previous variable

= new variable forthcoming, not previously collected

| Variable | Historical | Wave 1 | Wave 2 | Wave 3 | Wave 4 | Wave 5 |
| --- | --- | --- | --- | --- | --- | --- |
| Cognitive ability |  |  |  |  |  |  |
| Moray House Test No. 12 (MHT)^1^ |  |  |  |  |  |  |
| Mini-Mental State Examination (MMSE)^2^ |  |  |  |  |  |  |
| Wechsler Memory Scale III^UK^ - Logical Memory I & II^3^ |  |  |  |  |  |  |
| Wechsler Memory Scale III^UK^ - Spatial Span^3^ |  |  |  |  |  |  |
| Wechsler Memory Scale III^UK^ - Verbal Paired Associates ^3^ |  |  |  |  |  |  |
| Wechsler Memory Scale III^UK^ - Backward digit span^3^ |  |  |  |  |  |  |
| Wechsler Adult Intelligence Scale III^UK^ - Symbol Search^4^ |  |  |  |  |  |  |
| Wechsler Adult Intelligence Scale III^UK^ - Digit Symbol Coding^4^ |  |  |  |  |  |  |
| Wechsler Adult Intelligence Scale III^UK^ - Matrix Reasoning^4^ |  |  |  |  |  |  |
| Wechsler Adult Intelligence Scale III^UK^ - Letter-Number Sequencing^4^ |  |  |  |  |  |  |
| Wechsler Adult Intelligence Scale III^UK^ - Block Design^4^ |  |  |  |  |  |  |
| Simple and Choice reaction time^5, 6^ |  |  |  |  |  |  |
| Inspection Time^7^ |  |  |  |  |  |  |
| Verbal Fluency^8^ |  |  |  |  |  |  |
| National Adult Reading Test (NART)^9^ |  |  |  |  |  |  |
| Wechsler Test of Adult Reading (WTAR)^10^ |  |  |  |  |  |  |
| Test of Premorbid Functioning (TOPF)^11^ |  |  |  |  |  |  |
| Trail Making Test (Part B)^12^ |  |  |  |  |  |  |
| Raven’s Progressive Matrices^13^ |  |  |  |  |  |  |
| ‘Frontal’ function tests (sub-sample n =90)^14^ |  |  |  |  |  |  |
| Financial literacy and competence^15^ |  |  |  |  |  |  |
| Social and demographic data |  |  |  |  |  |  |
| Age (in days) |  |  |  |  |  |  |
| Marital status (single/married/cohabiting/divorced/widowed/other) |  |  |  |  |  |  |
| Living arrangement (living alone/not alone) |  |  |  |  |  |  |
| Current address |  |  |  |  |  |  |
| Residence type (home owned/rented/residential/nursing/hospital/other) |  |  |  |  |  |  |
| Place of Birth |  |  |  |  |  |  |
| Childhood address and overcrowding/deprivation details  (number of rooms and occupants in home, number sharing toilet, toilet indoor/outdoor) |  |  |  |  |  |  |
| Schools attended |  |  |  |  |  |  |
| Education  (years of education, age at leaving school, highest qualification obtained, lifelong learning experiences) |  |  |  |  |  |  |
| Main occupation of self (and spouse if female) |  |  |  |  |  |  |
| Socio-economic status (SES) coding^16^ |  |  |  |  |  |  |
| Retirement (age, reason, ever returned to work) |  |  |  |  |  |  |
| Current employment status, volunteering and caring (USP)^17^ |  |  |  |  |  |  |
| Questionnaires |  |  |  |  |  |  |
| Your Family (Occupation, education and survival of parents; birth place of parents and grandparents; year of birth, sex, education and occupation of children) |  |  |  |  |  |  |
| Lifetime home addresses and lifetime occupations of self and father (geographers) |  |  |  |  |  |  |
| World Health Organisation Quality of Life (WHOQOL-BREF)^18^ |  |  |  |  |  |  |
| Hospital Anxiety and Depression Scale (HADS)^19^ |  |  |  |  |  |  |
| International Personality Item Pool Five Factor Personality (IPIP)^20,,21^ |  |  |  |  |  |  |
| NEO Five Factor Inventory (NEO-FFI)^22^ |  |  |  |  |  |  |
| Satisfaction With Life Scale (SWLS)^23^ |  |  |  |  |  |  |
| Warwick-Edinburgh Mental Wellbeing Scale (WEMWBS)^24^ |  |  |  |  |  |  |
| LOT-R (Life Orientation Test-Revised)^25^ |  |  |  |  |  |  |
| Sense of Coherence (adapted from the Sense of Coherence Scale)^26^ |  |  |  |  |  |  |
| The Brief Resilience Scale^27^ |  |  |  |  |  |  |
| The Brief Serenity Scale^28^ |  |  |  |  |  |  |
| Dimensional Apathy Scale^29^ |  |  |  |  |  |  |
| Attitudes to Ageing Questionnaire (AAQ)^30^ |  |  |  |  |  |  |
| Support from others (current living arrangement, friends and relationships, presence of confidant and practical support/assistance, social interactions)^31-34^ |  |  |  |  |  |  |
| Loneliness (Wave 1: adapted from Social Support Questionnaire-Short Form ^34^; Waves 2-5: from the European Social Survey 3^35^ ; Wave 3: selected byAge UK^36^) |  |  |  |  |  |  |
| Perceived Social Support Scale^37^ |  |  |  |  |  |  |
| Personal and social wellbeing items (adapted from European Social Survey 3, section E)^35^ |  |  |  |  |  |  |
| Neighbourhood questions (adapted from the Neighbourhood Cohesion Scale)^38^ |  |  |  |  |  |  |
| Health Literacy (Rapid Estimate of Adult Literacy in Medicine^39^, Short Test of Functional Health Literacy in Adults ^40,41^, Newest Vital Sign^42^) |  |  |  |  |  |  |
| Food Frequency Questionnaire (FFQ) (Wave 1: Scottish Collaborative Group FFQ [version 7.0]^43^ ; Wave 4: EPIC-Norfolk FFQ ^44,45^) |  |  |  |  |  |  |
| Fruit and vegetable intake^46^ |  |  |  |  |  |  |
| Physical activity level and days per month of sport and exercise^47^ |  |  |  |  |  |  |
| Intellectual and social activities (level of participation)^48,49^ |  |  |  |  |  |  |
| Retrospective lifetime activity participation (sport and exercise, intellectual and social activities^47,49,50-53^ |  |  |  |  |  |  |
| Bilingualism^54^ |  |  |  |  |  |  |
| Self-reported memory problems |  |  |  |  |  |  |
| Sleep quality and pattern (adapted from the Pittsburgh Sleep Quality Index)^55^ |  |  |  |  |  |  |
| Daytime and nocturnal urination (adapted from International Prostate Symptom Score)^56^ |  |  |  |  |  |  |
| Understanding sedentary patterns questions |  |  |  |  |  |  |
| General self-efficacy scale^57^ |  |  |  |  |  |  |
| Social desirability scale^58^ |  |  |  |  |  |  |
| Selection, optimisation and compensation questions (adapted from Selection, optimisation and compensation (SOC) short version)^59^ |  |  |  |  |  |  |
| Sedentary behaviour diary |  |  |  |  |  |  |
| Musical experience and expertise |  |  |  |  |  |  |
| Physical and Medical |  |  |  |  |  |  |
| Self-rated health (Waves 2-4: general, Wave 4: physical and emotional)^60^ |  |  |  |  |  |  |
| Townsend Disability Scale^61,62^ |  |  |  |  |  |  |
| Incidence of falls and number requiring medical attention^63,64^ |  |  |  |  |  |  |
| Salt intake |  |  |  |  |  |  |
| Smoking history (current/ex/never, age at stopping, number cigarettes smoked per day) |  |  |  |  |  |  |
| Alcohol consumption (alcohol type and units per week) |  |  |  |  |  |  |
| Self-reported history of disease (blood pressure, diabetes, high cholesterol, cardiovascular disease, blood circulation, stroke, cancer, thyroid disorder, dementia, Parkinson’s Disease, arthritis, allergies, other) |  |  |  |  |  |  |
| Family history of disease (Waves 1-5: cardiovascular and cerebrovascular disease; Waves 2-5: dementia and Parkinson’s disease) |  |  |  |  |  |  |
| Current medications |  |  |  |  |  |  |
| Menopause (age, hysterectomy/oophorectomy, hormone replacement therapy use) |  |  |  |  |  |  |
| Height (cm) |  |  |  |  |  |  |
| Weight (kg) |  |  |  |  |  |  |
| BMI |  |  |  |  |  |  |
| Demi-Span (cm) |  |  |  |  |  |  |
| Head circumference (cm) |  |  |  |  |  |  |
| Facial symmetry photographs |  |  |  |  |  |  |
| Dentition (number of teeth/year lost) |  |  |  |  |  |  |
| Visual acuity at 6 metres measured by Snellen chart (corrected and uncorrected) |  |  |  |  |  |  |
| Hearing (self-reported and measured^65^ with HearCheck screener at 1000Hz and 3000Hz) |  |  |  |  |  |  |
| Systolic and diastolic blood pressure (sitting and standing) |  |  |  |  |  |  |
| Ankle-brachial pressure index (ABPI) |  |  |  |  |  |  |
| Lung Function (peak expiratory flow rate, forced expiratory volume in 1 second, forced vital capacity) |  |  |  |  |  |  |
| Grip strength of left and right hand (kg) |  |  |  |  |  |  |
| Time to walk 6 metres (in seconds) |  |  |  |  |  |  |
| Chair stand test |  |  |  |  |  |  |
| Stand tests (side-by-side, semi-tandem, tandem) |  |  |  |  |  |  |
| Room temperature during stand and chair stand tests |  |  |  |  |  |  |
| Bioelectrical impedance analysis |  |  |  |  |  |  |
| Sedentary behaviour pattern analysis by electronic activity monitor |  |  |  |  |  |  |
| Biochemistry and haematology analysis |  |  |  |  |  |  |
| Haemoglobin |  |  |  |  |  |  |
| Red cell count |  |  |  |  |  |  |
| Haematocrit |  |  |  |  |  |  |
| Mean cell volume |  |  |  |  |  |  |
| White cell count |  |  |  |  |  |  |
| Neutrophil count |  |  |  |  |  |  |
| Lymphocyte count |  |  |  |  |  |  |
| Monocyte count |  |  |  |  |  |  |
| Eosinophil count |  |  |  |  |  |  |
| Basophil count |  |  |  |  |  |  |
| Platelet count |  |  |  |  |  |  |
| Prothrombin time (PT) |  |  |  |  |  |  |
| PT ratio |  |  |  |  |  |  |
| Activated partial thromboplastin time (APTT) |  |  |  |  |  |  |
| APTT ratio |  |  |  |  |  |  |
| International Normalised Ratio (INR) Warfarin |  |  |  |  |  |  |
| Fibrinogen |  |  |  |  |  |  |
| D-Dimer |  |  |  |  |  |  |
| Ferritin |  |  |  |  |  |  |
| Vitamin B12 |  |  |  |  |  |  |
| Serum folate |  |  |  |  |  |  |
| Red cell folate |  |  |  |  |  |  |
| Urea |  |  |  |  |  |  |
| Creatinine |  |  |  |  |  |  |
| Estimated glomerular filtration rate (eGFR) |  |  |  |  |  |  |
| Sodium |  |  |  |  |  |  |
| Potassium |  |  |  |  |  |  |
| Albumin |  |  |  |  |  |  |
| Calcium |  |  |  |  |  |  |
| Cholesterol |  |  |  |  |  |  |
| Triglyceride |  |  |  |  |  |  |
| High density lipoprotein (HDL) cholesterol |  |  |  |  |  |  |
| Low density lipoprotein (LDL) cholesterol |  |  |  |  |  |  |
| Cholesterol: HDLC Ratio |  |  |  |  |  |  |
| Glycated haemoglobin (HbA1c) |  |  |  |  |  |  |
| Urate |  |  |  |  |  |  |
| Iron |  |  |  |  |  |  |
| Transferrin |  |  |  |  |  |  |
| Transferrin saturation (%) |  |  |  |  |  |  |
| C-reactive protein (CRP) |  |  |  |  |  |  |
| Thyroid stimulating hormone (TSH) |  |  |  |  |  |  |
| Thyroxine (Wave 1: Total T3; Waves 1-4: Free T4) |  |  |  |  |  |  |
| Sex hormone-binding globulin (SHBG) |  |  |  |  |  |  |
| Free androgen (FA) index |  |  |  |  |  |  |
| Testosterone |  |  |  |  |  |  |
| Urine Albumin |  |  |  |  |  |  |
| Urine Microalbumin |  |  |  |  |  |  |
| Urine Creatinine |  |  |  |  |  |  |
| Inflammation markers |  |  |  |  |  |  |
| High sensitivity CRP (hsCROP) |  |  |  |  |  |  |
| Von Willbrand Factor (VWF) |  |  |  |  |  |  |
| Interleukin 6 (IL-6) |  |  |  |  |  |  |
| Vascular endothelial growth factor (VEGF) |  |  |  |  |  |  |
| Intercellular adhesion molecule 1 (ICAM-1) |  |  |  |  |  |  |
| N-terminal prohormone of brain natriuretic peptide (NT-proBNP) |  |  |  |  |  |  |
| High sensitivity-Troponin T (hs-Troponin T) |  |  |  |  |  |  |
| Gamma-glytamyl transpeptidase (gGT) |  |  |  |  |  |  |
| Cystatin C |  |  |  |  |  |  |
| Brain derived neurotrophic facor (BDNF) |  |  |  |  |  |  |
| Lipoprotein A (LP(a)) |  |  |  |  |  |  |
| Tissue plasminogen activator (tPA) |  |  |  |  |  |  |
| Vitamin D |  |  |  |  |  |  |
| Apolipoprotein A1 (Apo A1) and Apolipoprotein (ApoB) |  |  |  |  |  |  |
| Leptin |  |  |  |  |  |  |
| Adiponectin |  |  |  |  |  |  |
| Oxidative Stress |  |  |  |  |  |  |
| Urinary-8 Isoprostane |  |  |  |  |  |  |
| Trolox equivalent antioxidant capacity (TEAC) |  |  |  |  |  |  |
| DNA damage |  |  |  |  |  |  |
| Antioxidant vitamins |  |  |  |  |  |  |
| Blood Brain Barrier |  |  |  |  |  |  |
| S100 beta |  |  |  |  |  |  |
| Saliva analysis |  |  |  |  |  |  |
| Cortisol (subsample of 90 participants) |  |  |  |  |  |  |
| DNA (if unable to obtain blood sample only) |  |  |  |  |  |  |
| Immunolgy |  |  |  |  |  |  |
| Cytomegalovirus (CMV) serostatus and titre |  |  |  |  |  |  |
| iPSC stem cell reprogramming |  |  |  |  |  |  |
| Peripheral blood mononuclear cell (PBMC) samples taken |  |  |  |  |  |  |
| Brain tissue analysis |  |  |  |  |  |  |
| Pre-mortem consent |  |  |  |  |  |  |
| Genetic analysis |  |  |  |  |  |  |
| Apolipoprotein E (APOE) genoptyping to determine APOE E4 allele status^66^ |  |  |  |  |  |  |
| Genome-wide genotyping |  |  |  |  |  |  |
| Whole genome sequencing |  |  |  |  |  |  |
| Telomere length |  |  |  |  |  |  |
| DNA methylation^67,68^ |  |  |  |  |  |  |
| Transcriptome-wide gene expression |  |  |  |  |  |  |
| Brain magnetic resonance imaging (MRI)^69^ |  |  |  |  |  |  |
| Diffusion tensor (DT-MRI) derived variables of long-range tracts |  |  |  |  |  |  |
| Whole brain structural connectome |  |  |  |  |  |  |
| White matter hyperintensity volume |  |  |  |  |  |  |
| Stroke lesion volume |  |  |  |  |  |  |
| Intracranial volume |  |  |  |  |  |  |
| Hippocampal volume |  |  |  |  |  |  |
| Hippocampal shape^70^ |  |  |  |  |  |  |
| Total brain tissue volume |  |  |  |  |  |  |
| Normal appearing white matter volume |  |  |  |  |  |  |
| Grey matter volume |  |  |  |  |  |  |
| Separate and total ventricular volume |  |  |  |  |  |  |
| Visual atrophy and lesion rating scales |  |  |  |  |  |  |
| Neuroradiological report |  |  |  |  |  |  |
| Cortical thickness (CIVET)^71^ |  |  |  |  |  |  |
| Desikan-Killiany Atlast Parcellation (FreeSurfer) – volume and surface area^72,73^ |  |  |  |  |  |  |
| Carotid Doppler ultrasound |  |  |  |  |  |  |
| Velocities (common carotid artery (CCA), internal carotid artery (ICA), external carotid artery (ECA), vertebral arteries (VA)) |  |  |  |  |  |  |
| Stenosis |  |  |  |  |  |  |
| Intima-media thickness (CCA, bulb) |  |  |  |  |  |  |
| Retinal imaging |  |  |  |  |  |  |
| Semi-automated measures of microvascular caliber, branching geometry, fractal dimension and tortuosity by Vascular Assessment and Measurement Platform for Images (VAMPIRE)^74^ and Singapore I Vessel Assessment (SIVA)^75^ |  |  |  |  |  |  |
| Linkages |  |  |  |  |  |  |
| Date and cause of death |  |  |  |  |  |  |
| Hospital discharge diagnosis |  |  |  |  |  |  |
| Dementia diagnosis |  |  |  |  |  |  |
| Medical prescription data |  |  |  |  |  |  |

**Supplementary References (for Supplementary Table 4)**

1. Scottish Council for Research in Education. *The trend of Scottish intelligence*. London, UK: University of London Press, 1949.
2. Folstein MF, Folstein SE, McHugh PR. Mini-Mental State: A practical method for grading the cognitive state of patients for the clinician. *J Psychiat Res* 1975; 12:189-198.
3. Wechsler D. *WMS-IIIUK administration and scoring manual*. London, UK: Psychological Corporation, 1998.
4. Wechsler D. *WAIS-IIIUK administration and scoring manual*. London, UK: Psychological Corporation, 1998.
5. Cox BD, Huppert FA, Whichelow MJ. *The health and lifestyle survey: seven years on Aldershot*. UK, Dartmouth, 1993.
6. Deary IJ, Der G, Ford G. Reaction times and intelligence differences: a population-based cohort study. *Intelligence* 2001; 29:389-399.
7. Deary IJ, Simonotto E, Meyer M *et al*. The functional anatomy of inspection time: an event-related fMRI study. *Neuroimage* 2004; 22:1466-1479.
8. Lezak M. *Neuropsychological testing*. Oxford: Oxford University Press, 2004.
9. Nelson HE, Willison JR. National Adult Reading Test (NART) Test Manual (Part II). Windsor, UK: NFER-Nelson, 1991.
10. Holdnack JA. *WTAR: Wechsler Test of Adult Reading manual.* San Antonio, TX: Psychological Corporation, 2001.
11. Wechsler D. *Test of Premorbid Functioning – UK Version (TOPF UK).* London, UK: Psychological Corporation, 2011.
12. Reitan RM. Validity of the Trail Making test as an indicator of organic brain damage. *Percept Mot Skills* 1958; 8:271-276.
13. Raven JC, Court JH, Raven J. *Manual for Raven’s progressive matrices and vocabulary scales.* London, England: H. K. Lewis, 1977.
14. Cox SR, MacPherson SE, Ferguson KJ *et al*. Correlational structure of ‘frontal’ tests and intelligence tests indicates two components with asymmetrical neurostructural correlates in old age. *Intelligence* 2014; 94-106.
15. Gerstenecker A., Eakin A., Triebel K, *et al*. Age and Education Corrected Older Adult Normative Data for a Short Form Version of the Financial Capacity Instrument. *Psychol Assess* 2016; 28:737-749.
16. General Register Office. *Census 1951: classification of occupations*. London, UK: Her Majesty’s Stationary Office, 1956.
17. West of Scotland Twenty-07 Study Health in the Community Interview Schedules. Website title: [http://2007study.sphsu.mrc.ac.uk/W5-all-R-and-L-FandO_2012_09.pdf]
18. Group WHOQOL: Development of the World Health Organization WHOQOL-BREF Quality of Life Assessment. *Psychol Med* 1998, 28:551–558.
19. Zigmond AS, Snaith RP. The Hospital Anxiety and Depression Scale. *Acta Psyschiatr Scanda* 1983; 67:361-370.
20. Goldberg LR. *A broad-bandwidth public domain, personality inventory measuring the lower-level facets of several five-factor models*. In I. Mervielde, I. J. Deary, F. de Fruyt, & F. Ostendorf (Eds.). Personality psychology in Europe (Vol. 7, pp. 7–28). Tilburg: Tilburg University Press.
21. Website title: [http://ipip.ori.org/].
22. Costa PT, McCrae RR. *Revised NEO personality inventory (NEO PI-R) and NEO five-factor inventory (NEO-FFI) professional manual*. Odessa, FL: Psychological Assessment Resources, 1992.
23. Diener E, Emmons RA, Larsen RJ, Griffin S. The Satisfaction With Life Scale. *J Pers Assess* 1985; 49:71-75.
24. Tennant R, Hiller L, Fishwick R, Platt S, Joseph S, *et al*. The Warwick- Edinburgh Mental Well-being Scale (WEMWBS): development and UK validation. *Health Qual Life Outcomes* 2007; 5:63
25. Scheier MF, Carver CS, Bridges MW. Distinguishing optimism from neuroticism (and trait anxiety, self-mastery, self-esteem): a re-evaluation of the Life Orientation Test. *J Pers Soc Psychol* 1994; 67:1063–1078.
26. Lundberg O, Nystrom Peck M. A simplified way of measuring sense of coherence: experiences from a population survey in Sweden. *Eur J Public Health* 1995; 5: 56-59.
27. Smith BW, Dalen J, Wiggins K, Tooley E, Christopher P, Bernard J. The Brief Resilience Scale: assessing the ability to bounce back. *Int J Behav Med* 2008; 15:194-200.
28. Kreitzer MJ, Gross CR, Waleekhachonloet O, Reilly-Spong M, Byrd M. The Brief Serenity Scale: a psychometric analysis of a measure of spirituality and well-being. *J Holist Nurs* 2009; 27:7-16.
29. Radakovic R, Abrahams S. Developing a new apathy measurement scale: Dimensional Apathy Scale. *Psychiatry Rese* 2014; 219: 658-663.
30. Laidlaw K, Power MJ, Schmidt S. The Attitudes to Ageing Questionnaire (AAG): development and psychometric properties. *Int J Geriatr Psychiatry* 2007; 22:367-379.
31. Seeman TE, Berkman LF. Structural characteristics of social networks and their relationship with social support in the elderly: who provides support. *Soc Sci Med* 1988, 26:737-749.
32. Bassuk SS, Glass TA, Berkman LF. Social disengagement and incident cognitive decline in community dwelling elderly persons. *Ann Intern Med* 1999, 131:165-173.
33. Sherbourne CD, Stewart AL. The MOS Social Support Survey. *Soc Sci Med* 1991, 32:705-714
34. Sarason IG, Sarason BR, Shearin EN, Pierce GR. A Brief Measure of Social Support: Practical and Theoretical Implications. *J Soc Pers Relat* 1987, 4:497-510.
35. ESS Round 3: European Social Survey (2016): ESS-3 2006 Documentation Report. Edition 3.6. Bergen, European Social Survey Data Archive, NSD - Norwegian Centre for Research Data for ESS ERIC.
36. Victor CR, Bowling A. A longitudinal analysis of loneliness among older people in Great Britain. *J Psychol* 2012; 146: 313-331.
37. Shields MA, Wheatley Price S. Exploring the economic and social determinants of psychological well-being and perceived social support in England. *J R Stat Soc Ser A Stat Soc* 2005; 168:513-537.
38. Buckner JC. The development of an instrument to measure neighbourhood cohesion. *Am J Community Psychol* 1988; 16:771-791.
39. Davis TC, Long SW, Jackson RH *et al*. Rapid estimate of adult literacy in medicine: a shortened screening instrument. *Fam Med* 1993; 25: 613-625.
40. Baker DW, Williams MV, Parker RM, Gazmararian JA, Thompson JA, Huang J. Development of a brief test to measure functional health literacy. *Patient Educ Couns* 1999; 38: 33-42.
41. Von Wagner C, Steptoe A, Wolf MS, Wardle J. Functional health literacy and health-promoting behaviour in a national sample of British adults. *J Epidemiol Community Health* 2007; 61: 1086-1090.
42. Weiss BD, Mays MZ. Martz W, *et al*. Quick assessment of literacy in primary care: the newest vital sign. *Ann Fam Med* 2005; 3: 514-522.
43. Masson LF, McNeill G, Tomany JO, *et al*. Statistical approaches for assessing the relative validity of a food-frequency questionnaire: use of correlation coefficients and the kappa statistic*. Public Health Nutr* 2003, 6:313-321.
44. Mulligan AA, Luben RN, Bhaniani A, *et al*. A new tool for converting food frequency questionnaire data into nutrient and food group values: FETA research methods and availability. *BMJ Open* 2014; 4:e004503.
45. Website title: [<http://www.srl.cam.ac.uk/epic/nutmethod/FFQ.shtml>].
46. Cappuccio FP, Rink E, Perkins-Porras L, *et al*. Estimation of fruit and vegetable intake using a two-item dietary questionnaire: a potential tool for primary health care
    workers. *Nutr Metab Cardiovasc Dis* 2003; 13: 12–9.
47. Hirvensalo M, Lampinen P, Rantanen T. Physical exercise in old age: an eight-year- follow-up study on involvement, motives, and obstacles among persons age 65–84. *J Aging Phys Act* 1998, 6:157-168.
48. Wilson RS, Bennett DA, Bienias JL, *et al*. Cognitive activity and incident AD in a population-based sample of older persons. *Neurology* 2002, 59:1910-1914.
49. Hultsch DF, Hertzog C, Small BJ, Dixon RA. Use it or lose it: engaged lifestyle as a buffer of cognitive decline in aging? *Psychol Aging* 1999, 14:245-63.
50. Glass TA, de Leon CM, Marottoli RA & Berkman LF. Population based study of social and productive activities and as predictors of survival among elderly Americans. *BMJ* 1999; 319, 478-483.
51. Richards M, Hardy R & Wadsworth MEJ. Does active leisure protect cognition? Evidence from a national birth cohort. *Soc Sci Med* 2003; 56, 785-792.
52. Wilson RS, Barnes LL & Bennett DA. Assessment of lifetime participation in cognitively stimulating activities. Journal of Clinical and Experimental *Neuropsychology* 2003; 25, 634-642.
53. Wilson RS, Beckett LA, Barnes LL, Schneider JA, Bach J, Evans DA *et al*. Individual differences in rates of change in cognitive abilities of older persons. *Psych Aging* 2002; 17, 179-193.
54. Bak TH, Nissan JJ, Allerhand MM, Deary IJ. Does bilingualism influence cognitive aging? *Ann Neurol* 2014; 75: 959-963.
55. Buysse DJ, Reynolds CF, Monk TH, Berman SR, Kupfer DJ. The Pittsburgh Sleep Quality Index (PSQI): a new instrument for psychiatric research and practice. *Psychiatry Res* 1989; 28: 193-213.
56. Barry MJ, Fowler FJ, O’Leary MP *et al*. The American Urological Association symptom index for benign prostatic hyperplasia. The Measurement Committee of the American Urological Association. *J Urol* 1992: 148; 1549-57.
57. Schwarzer R., Jerusalem M. *Generalized Self-Efficacy scale*. In J. Weinman, S. Wright, & M. Johnston, Measures in health psychology: A user’s portfolio. Causal and control beliefs (pp. 35- 37). Windsor, England: NFER-NELSON, 1995.
58. Rudmin FW. Norwegian short-form of the Marlowe-Crowne Social Desirability Scale. *Scand J Psychol* 1999; 40: 229-233.
59. Baltes PB, Baltes MM, Freund AM, Lang FR. *The measurement of selection, optimisation, and compensation (SOC) by self-report: Technical report 1999*. Berlin: Max Planck Institute for Human Development, 1999.
60. Ware JE, Sherbourne CD. The MOS 36-Item Short-Form Health Survey (SF-36): 1. Conceptual framework and item selection. *Med Care* 1992; 30: 473-483.
61. Bond J, Carstairs V. *Services for the elderly: a survey if the characteristics and needs of a population of 5,000,000 old people (Scottish Home and Health Studies No. 42)* Edinburgh, Scotland: Scottish Home and Health Department, 1982.
62. Townsend P. *Poverty in the United Kingdom*. Hardmonsworth, England: Pelican, 1979.
63. Lamb SE, Jorstad-Stein EC, Hauer K, Becker C, Prevention of Falls Network Europe and Outcomes Consensus Group. Development of a common outcome data set for fall injury prevention trials: the Prevention of Falls Network Europe consensus. *J AM Geriatr Soc* 20015; 53:1618-22.
64. Hauer K, Lamb SE, Jorstad EC, Todd C, Becker C, PROFANE-Group. Systematic review of definitions and methods of measuring falls in randomised controlled fall prevention trials. *Age Ageing* 2006; 35:5-10.
65. Akeroyd M. Hearing questionnaire adapted from a postal survey by Stuart Gatehouse, IHR Scottish Section 2004.
66. Wenham PR, Price, WH, Blandell G. Apolipoprotein E genotyping by one-stage PCR. *Lancet* 1991; 337:1158-1159.
67. Hannum G, Guinney J, Zhao L *et al*. Genome-wide methylation profiles reveal quantitative views of human aging rates. *Mol Cell* 2013;49:359-67.
68. Horvath S. DNA methylation age of human tissues and cell types. *Genome Biol* 2013;14:R115.
69. Wardlaw JM, Bastin ME, Valdes Hernandez MC *et al*. Brain aging, cognition in youth and old age and vascular disease in the Lothian Birth Cohort 1936: rationale, design and methodology of the imaging protocol. *J Stroke* 2011; 6: 547-559.
70. Cox SR, Valdes Hernandez MC, Kim J *et al*. Associations between hippocampal morphology, diffusion characteristics, and salivary cortisol in older men. *Psychoneuroendocrinology* 2017, 78:151-158.
71. Karama S, Bastin ME, Murray C *et al*. Childhood cognitive ability accounts for associations between cognitive ability and brain cortical thickness in old age. *Mol Psychiatry* 2014; 19:555-9.
72. Desikan RS, Segonne F, Fischl B et al. An automated labelling system for subdividing the human cerebral cortex on MRI scans into gyral based regions of interest. NeuroImage 2006; 31:968-980.
73. Cox SR, Bastin ME, Ritchie SJ *et al*. Brain cortical characteristics of lifetime cognitive aging. *Brain Struct Funct* 2017; doi;https://doi.org/10.1007/s00429-017-1505-0.
74. Perez-Rovira A, MacGillivray T, Trucco E, *et al*. VAMPIRE: vessel assessment and measurement platform for images of the REtina. *Conf Proc IEEE Eng Med Biol Soc* 2011; 2011:3391-3394.
75. Lau QP, Lee ML, Hsu W, Wong TY. *The Singapore Eye Vessel Assessment System.* Ng EYK, Acharya UR, Campilo A, Suri JS, eds. Image Analysis and Modeling in Ophthalmology: Boca Raton, FL, US: CRC Press; 2014: 143-160.
